# Supplementary material for: Selecting likely causal risk factors from high-throughput experiments using multivariable Mendelian randomization
Source: Nat Commun. 2020 Jan 7;11:29. doi: 10.1038/s41467-019-13870-3 (PMC6946691; doi:10.1038/s41467-019-13870-3)
Supplement: Supplementary file 1 — Supplementary Information [file 41467_2019_13870_MOESM1_ESM.pdf]

Supplementary Information: Selecting likely causal risk factors from high-throughput experiments using multivariable Mendelian randomization

Verena Zuber[1,2], Johanna Maria Colijn[3,4], Caroline Klaver[3,4,5], and Stephen Burgess[1,6]

[1]MRC Biostatistics Unit, School of Clinical Medicine, University of Cambridge, UK
[2]Department of Epidemiology and Biostatistics, Imperial College London, UK
[3]Department of Epidemiology, Erasmus University Medical Center, Rotterdam, The Netherlands
[4]Department of Ophthalmology, Erasmus University Medical Center, Rotterdam, The Netherlands
[5]Department of Ophthalmology, Radboud University Medical Center, Nijmegen, The Netherlands
[6]MRC/BHF Cardiovascular Epidemiology Unit, School of Clinical Medicine, University of Cambridge, UK

Contents

1 Supplementary Figures **4**

2 Supplementary Tables **16**

3 Supplementary Methods **35**

List of Tables

1 Bias from the simulation study 20
2 Mean and standard deviation of the causal effect estimate from the simulation study: Setting A 21
3 Mean and standard deviation of the causal effect estimate from the simulation study: Setting B 22
4 Ranking of risk factors (top ten) for age-related macular degeneration including all variants 23
5 q-statistic for all genetic variants 24
6 Cook's distance for all genetic variants 25
7 Complete ranking of risk factors for age-related macular degeneration excluding outliers and influential points 26
8 q-statistic for $n = 145$ genetic variants excluding *LIPC, FUT2* and *APOE* 27
9 Cook's distance for $n = 145$ genetic variants excluding *LIPC, FUT2* and *APOE* 28
10 Parameter check: Prior probability 29
11 Parameter check: Prior variance 30
12 Ranking of risk factors for age-related macular degeneration using IVW-regression 31
13 Ranking of risk factors for age-related macular degeneration using Lars 32
14 Ranking of risk factors for age-related macular degeneration using Lasso 33
15 Ranking of risk factors for age-related macular degeneration using Elastic Net 34

List of Figures

1 Directed acyclic graph to illustrate the difference between total and direct effect 4
2 Genetic correlation between metabolite measurements based on the $n = 148$ genetic variants used as instrumental variables. . . . 5
3 Receiver operating characteristic curve for setting A including a samll number of risk factors ($d = 12$) 6

4 Receiver operating characteristic curve for setting B including a small number of risk factors ($d = 12$) 6
5 Causal effect estimates for setting A including a small number of risk factors ($d = 12$) 7
6 Causal effect estimates for setting B including a small number of risk factors ($d = 12$) 8
7 Receiver operating characteristic curve for setting A including a large number of risk factors ($d = 92$) 9
8 Receiver operating characteristic curve for setting B including a large number of risk factors ($d = 92$) 9
9 Causal effect estimate for setting A including a large number of risk factors ($d = 92$) 10
10 Causal effect estimate for setting B including a large number of risk factors ($d = 92$) 11
11 Genetic correlation between blood cell traits based on the $n = 2667$ genetic variants used as instrumental variables. 12
12 Receiver operating characteristic curve for setting A including ($d = 33$) risk factors 13
13 Receiver operating characteristic curve for setting B including ($d = 33$) risk factors 13
14 Causal effect estimate for setting A for the blood cell traits ($d = 33$) 14
15 Causal effect estimate for setting B for the blood cell traits ($d = 33$) 15
16 Diagnostic plots of the predicted associations with AMD against the observed associations with AMD 16
17 Scatterplot of associations of the top risk factors against associations with the outcome 17
18 Diagnostic plots (part 1) of the predicted associations with AMD against the observed associations with AMD after excluding the *LIPC, FUT2* and *APOE* gene regions 18
19 Diagnostic plots (part 2) of the predicted associations with AMD against the observed associations with AMD after excluding the *LIPC, FUT2* and *APOE* gene regions 19

1 Supplementary Figures

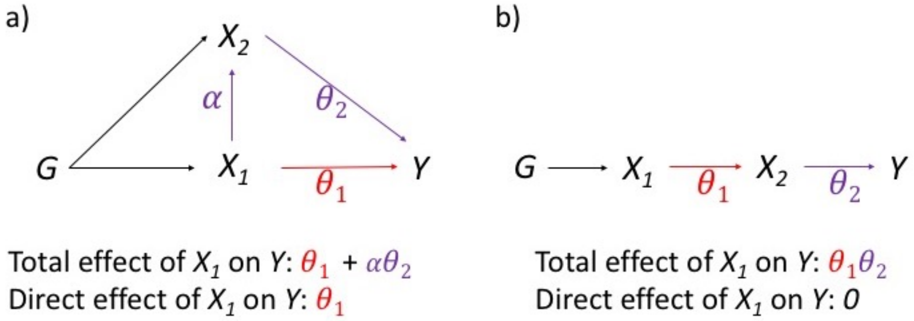

Supplementary Figure 1: Directed acyclic graph to illustrate the difference between total and direct effect in two scenarios: a) mediation effect, where the risk factor X_1 has a direct and an indirect effect via the mediator X_2 on the outcome Y and b) signalling cascade where the effect of X_1 on the outcome is entirely mediated by X_2.

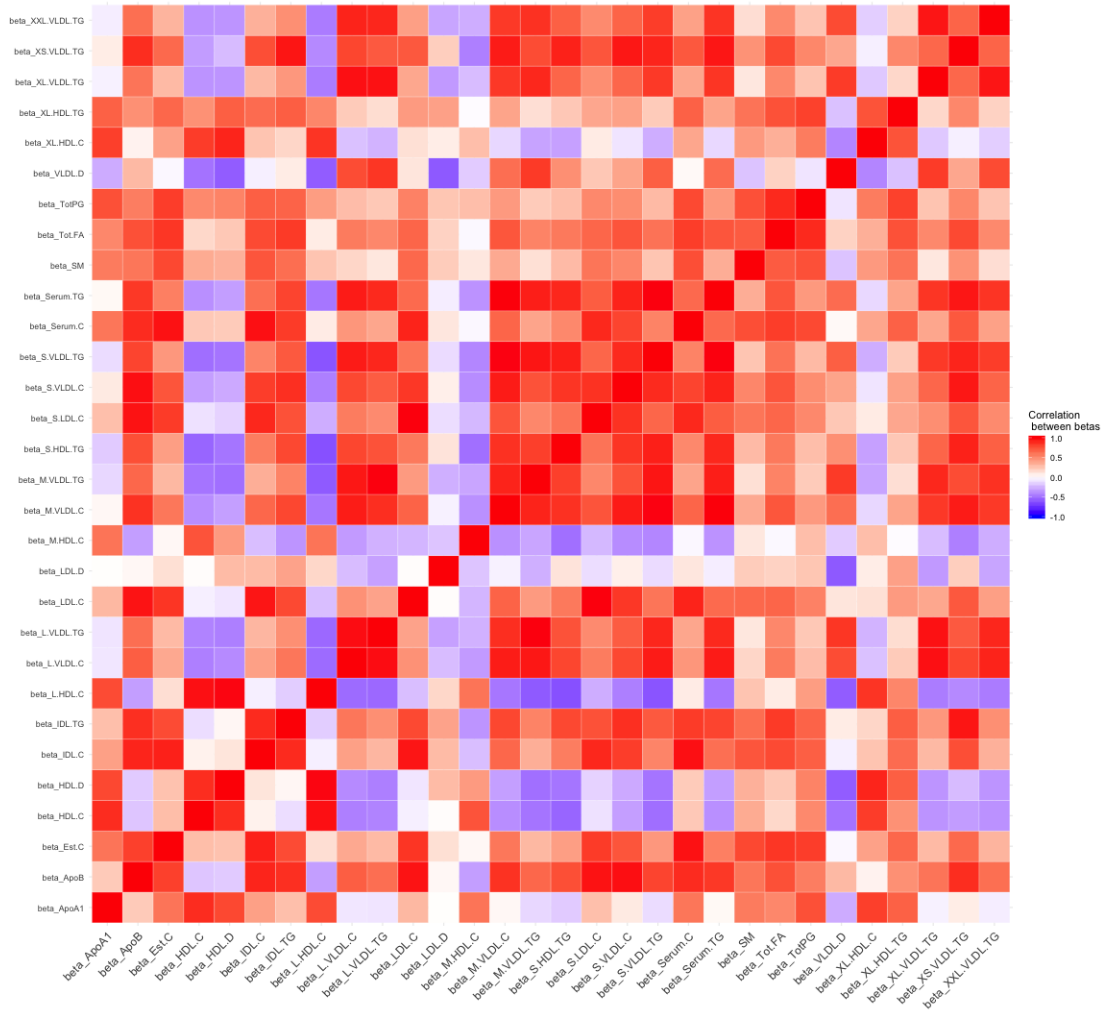

Supplementary Figure 2: Genetic correlation between metabolite measurements based on the $n = 148$ genetic variants used as instrumental variables.

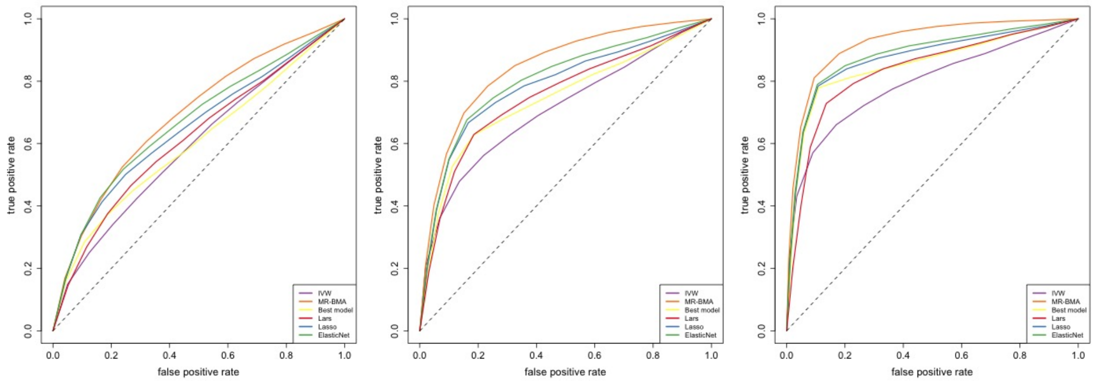

Supplementary Figure 3: Receiver operating characteristic (ROC) curve for setting A including a small number of risk factors ($d = 12$) of which four are true positive effects. Proportion of variance explained is set to 0.1 (left) 0.3 (middle) and 0.5 (right).

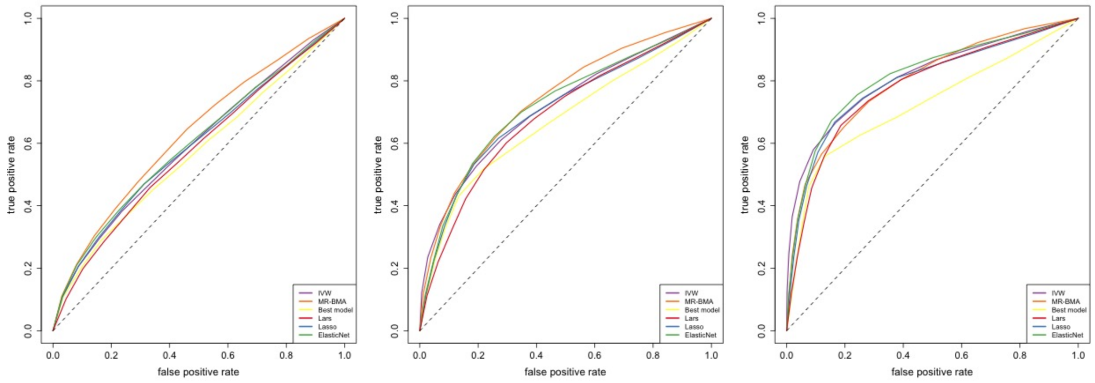

Supplementary Figure 4: Receiver operating characteristic (ROC) curve for setting B including a small number of risk factors ($d = 12$) of which eight are true positive effects (four postive and four negative effect direction). Proportion of variance explained is set to 0.1 (left) 0.3 (middle) and 0.5 (right).

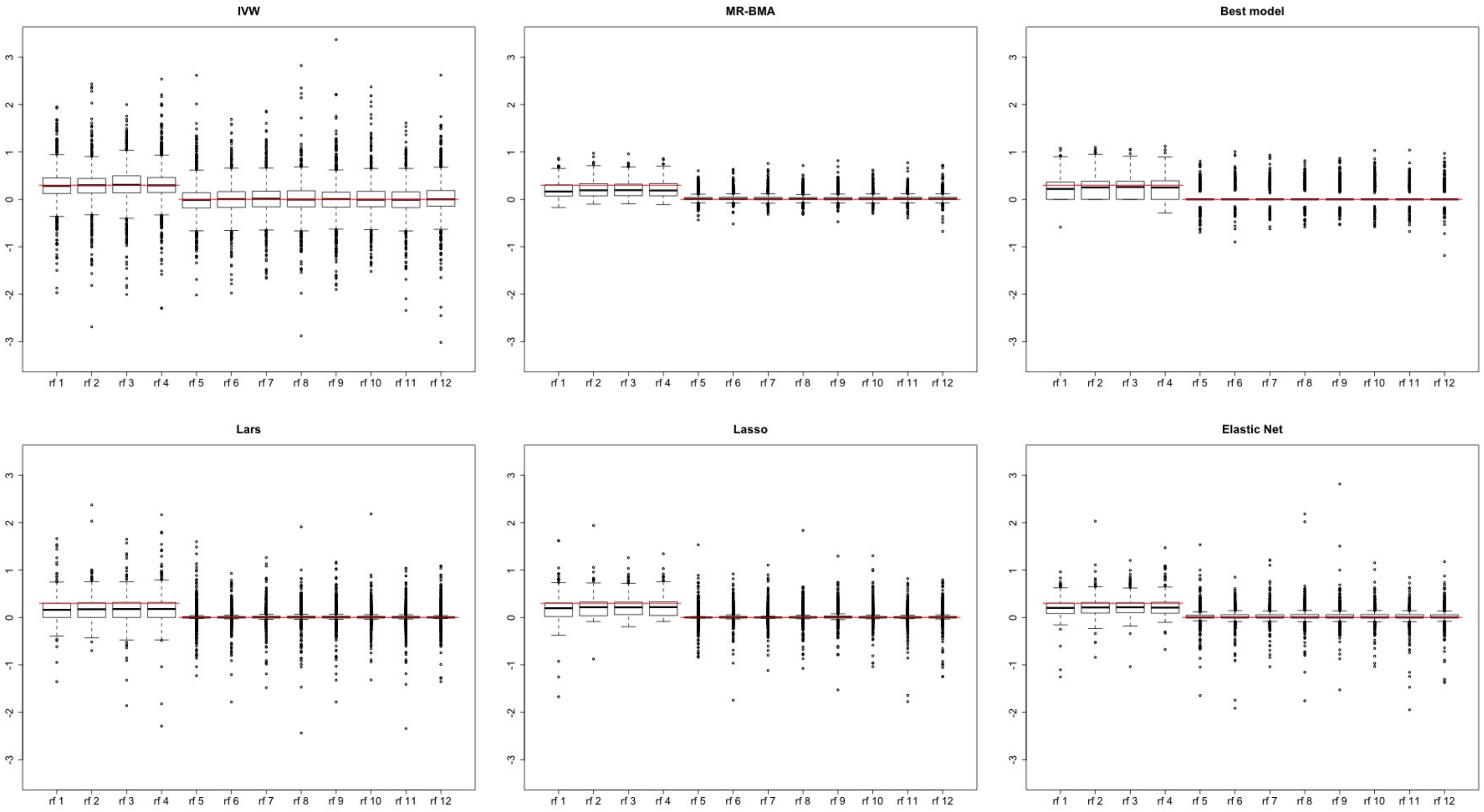

Supplementary Figure 5: Boxplots of the causal effect estimates for setting A including a small number of risk factors ($d = 12$) of which the first four are true positive effects. The true causal effects are marked in red. From top left to bottom right are the competing approaches: IVW, MR-BMA, best model, Lars, Lasso, and Elastic Net. Proportion of variance explained is set to 0.3.

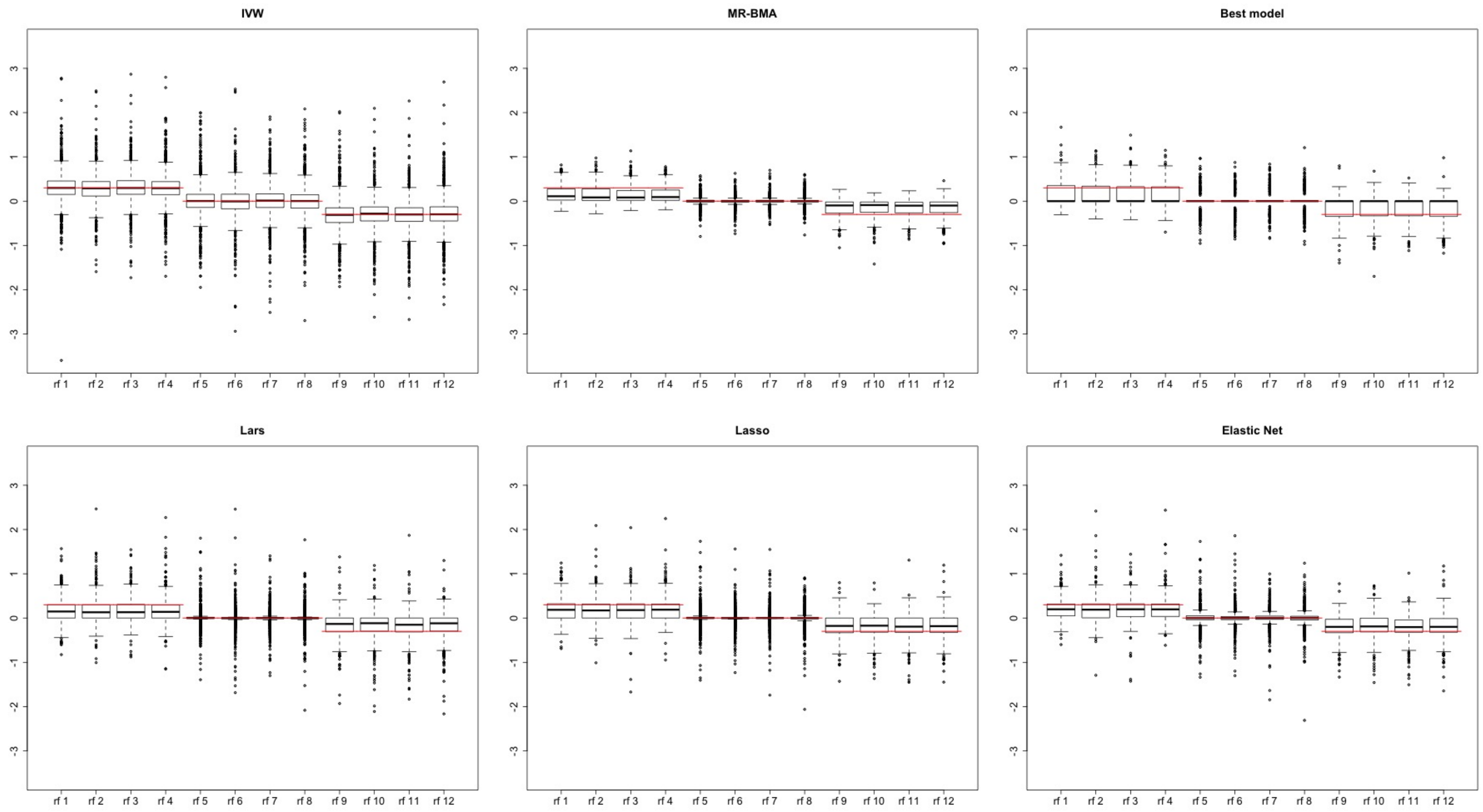

Supplementary Figure 6: Boxplots of the causal effect estimates for setting B including a small number of risk factors ($d = 12$) of which the first four have a positive and final four have a negative causal effect. The true causal effects are marked in red. From top left to bottom right are the competing approaches: IVW, MR-BMA, best model, Lars, Lasso, and Elastic Net. Proportion of variance explained is set to 0.3.

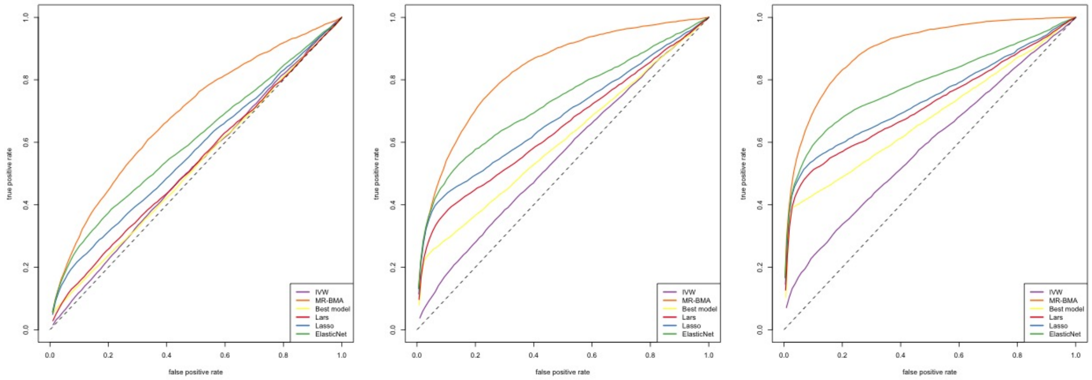

Supplementary Figure 7: Receiver operating characteristic (ROC) curve for setting A including a large number of risk factors ($d = 92$) of which four are true positive effects. Proportion of variance explained is set to 0.1 (left) 0.3 (middle) and 0.5 (right).

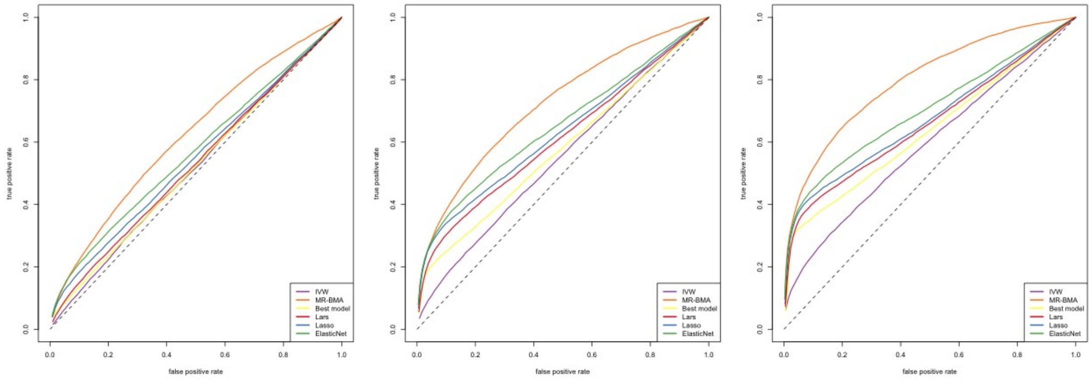

Supplementary Figure 8: Receiver operating characteristic (ROC) curve for setting B including a large number of risk factors ($d = 92$) of which eight are true positive effects (four postive and four negative effect direction). Proportion of variance explained is set to 0.1 (left) 0.3 (middle) and 0.5 (right).

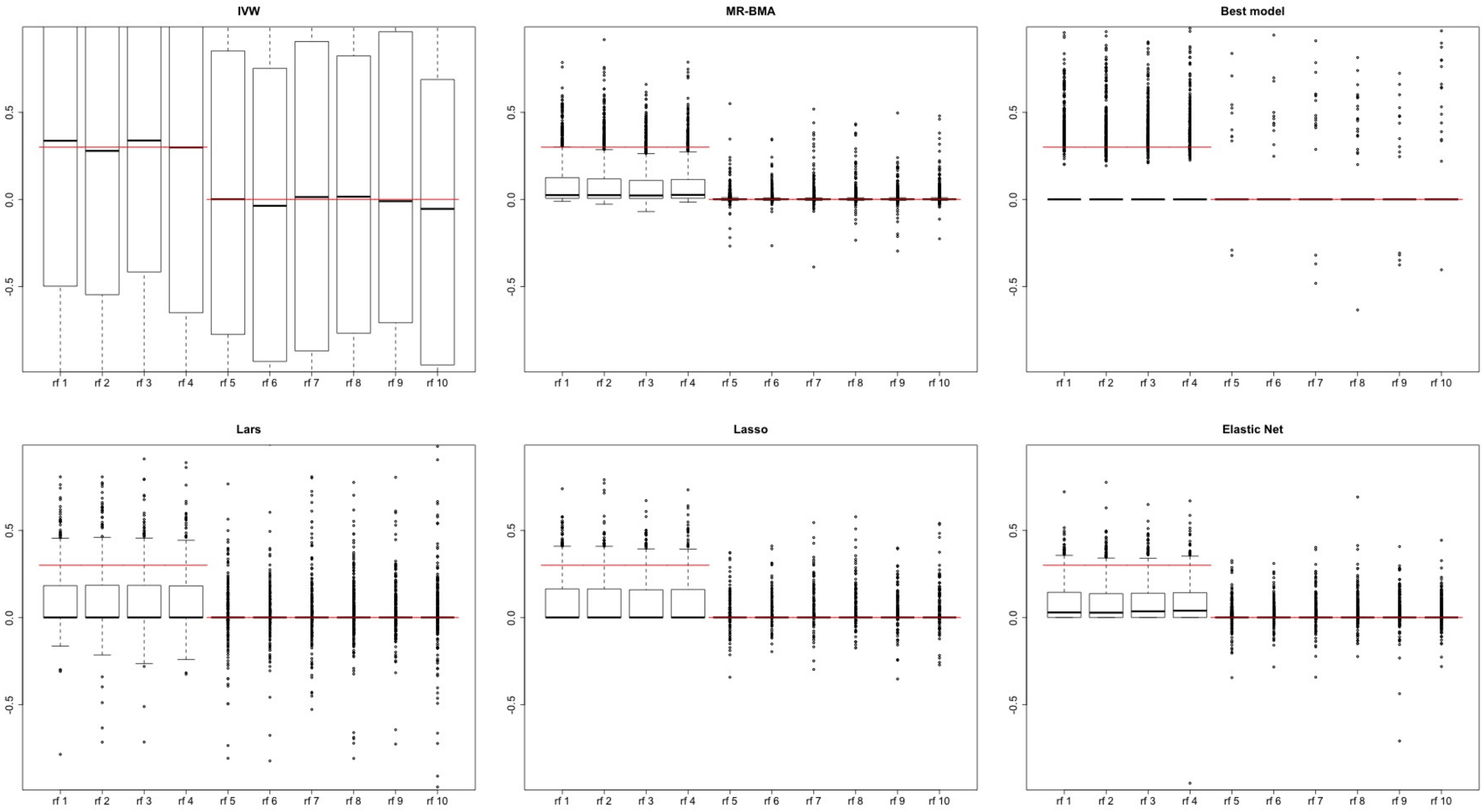

Supplementary Figure 9: Boxplots of the causal effect estimates for setting A including a large number of risk factors ($d = 92$) of which the first four are true positive effects. Risk factors 11 to 92 are omitted. The true causal effects are marked in red. From top left to bottom right are the competing approaches: IVW, MR-BMA, best model, Lars, Lasso, and Elastic Net. Proportion of variance explained is set to 0.3.

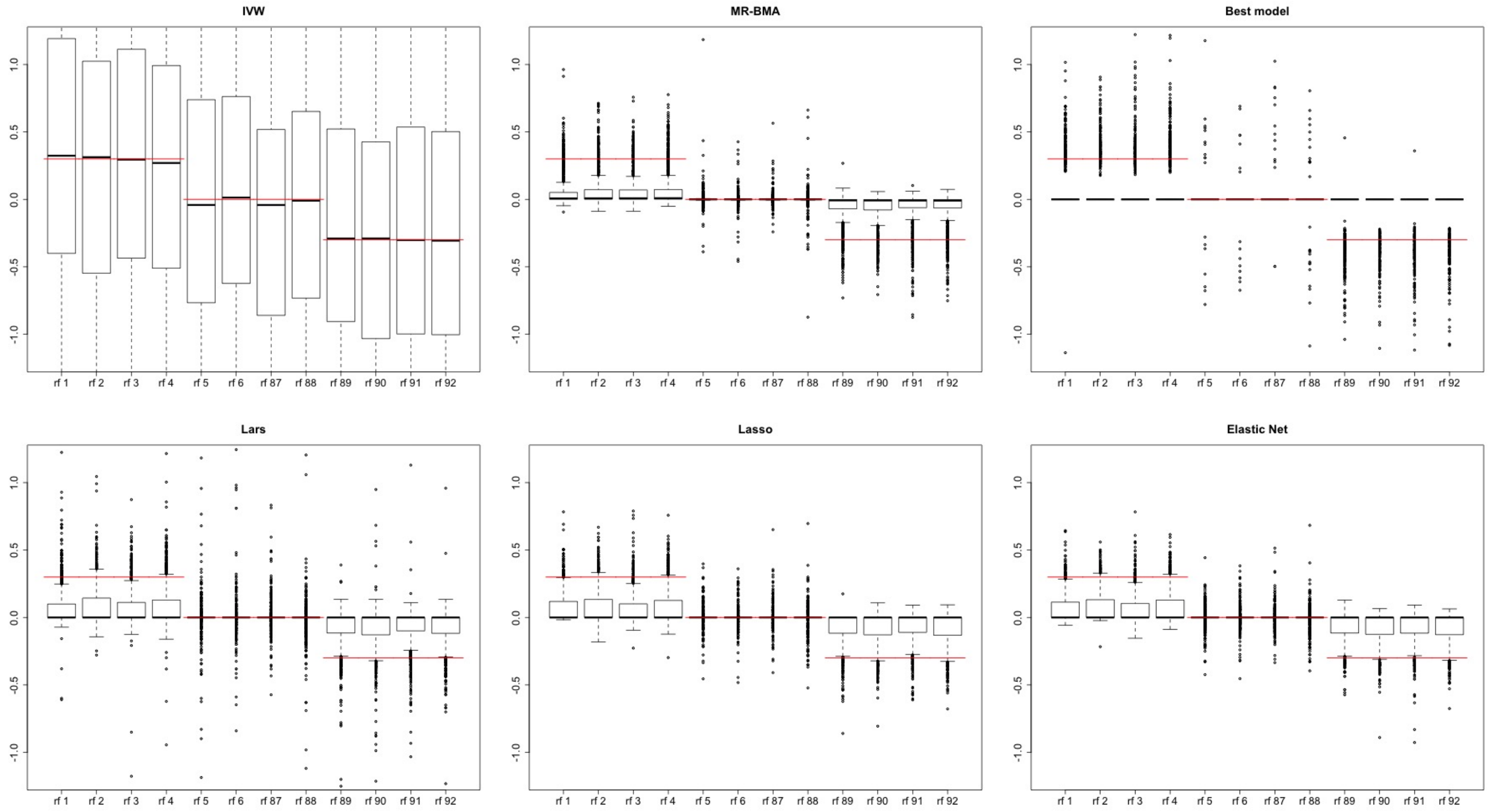

Supplementary Figure 10: Boxplots of the causal effect estimates for setting B including a large number of risk factors ($d = 92$) of which the first four have a positive and the final 4 have a negative causal effect. Risk factors 7 to 86 are omitted. The true causal effects are marked in red. From top left to bottom right are the competing approaches: IVW, MR-BMA, best model, Lars, Lasso, and Elastic Net. Proportion of variance explained is set to 0.3.

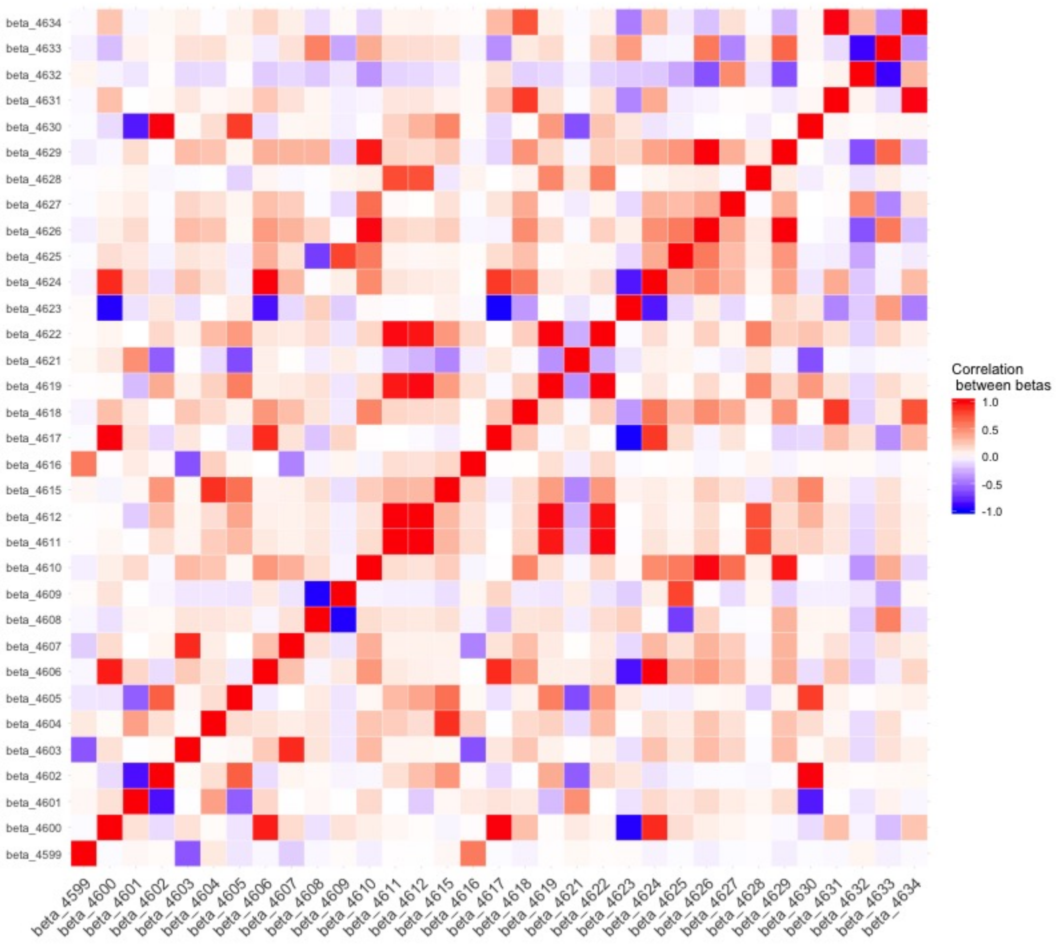

Supplementary Figure 11: Genetic correlation between blood cell traits based on the $n = 2667$ genetic variants used as instrumental variables.

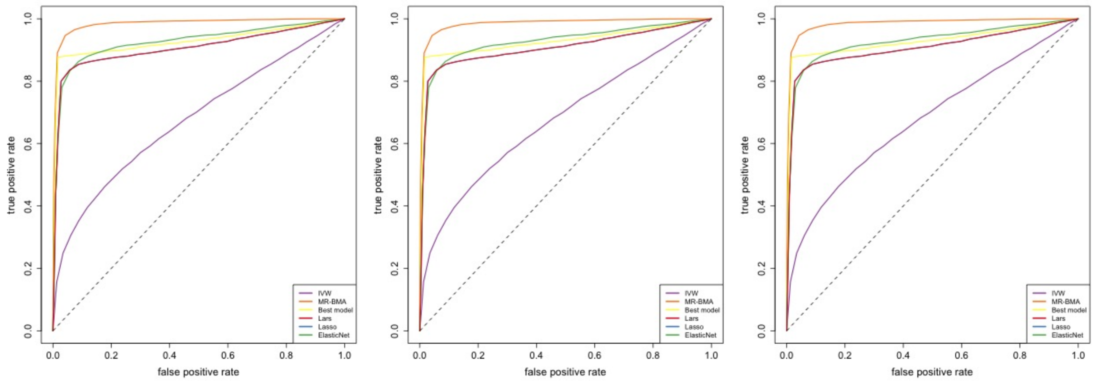

Supplementary Figure 12: Receiver operating characteristic (ROC) curve for setting A including ($d = 33$) blood cell traits as risk factors of which four are true positive effects. Proportion of variance explained is set to 0.1 (left) 0.3 (middle) and 0.5 (right).

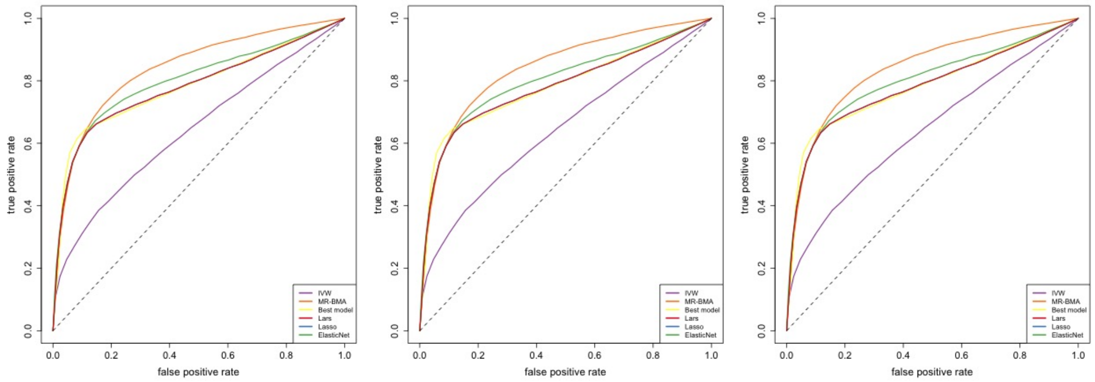

Supplementary Figure 13: Receiver operating characteristic (ROC) curve for setting B including ($d = 33$) blood cell traits as risk factors of which four have true positive effect and another four have true negative effect. Proportion of variance explained is set to 0.1 (left) 0.3 (middle) and 0.5 (right).

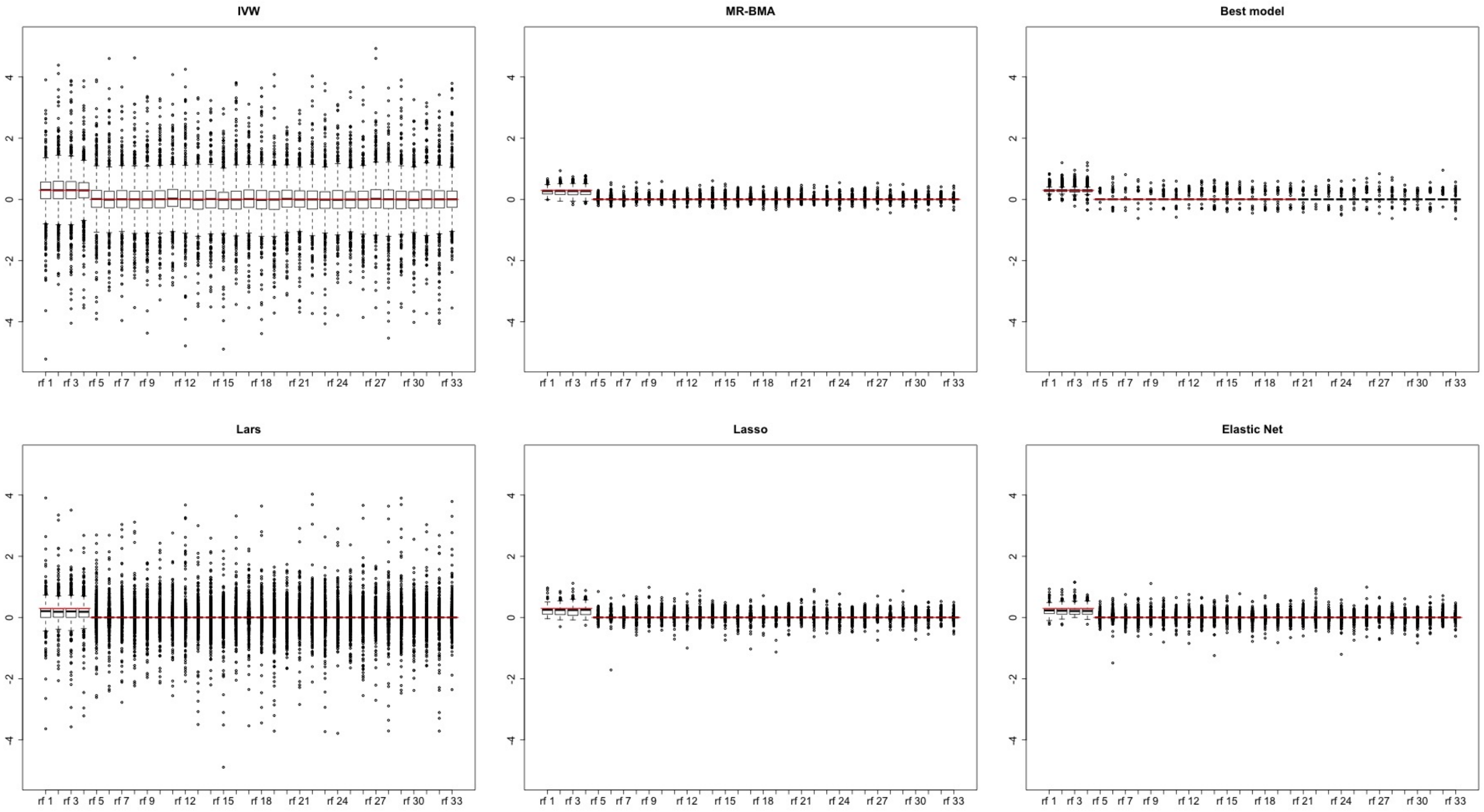

Supplementary Figure 14: Boxplots of the causal effect estimates for setting A for the blood cell traits ($d = 33$), of which the first four are true positive effects. The true causal effects are marked in red. From top left to bottom right are the competing approaches: IVW, MR-BMA, best model and Lars, Lasso and Elastic Net (all tuned with cross-validation). Proportion of variance explained is set to 0.3.

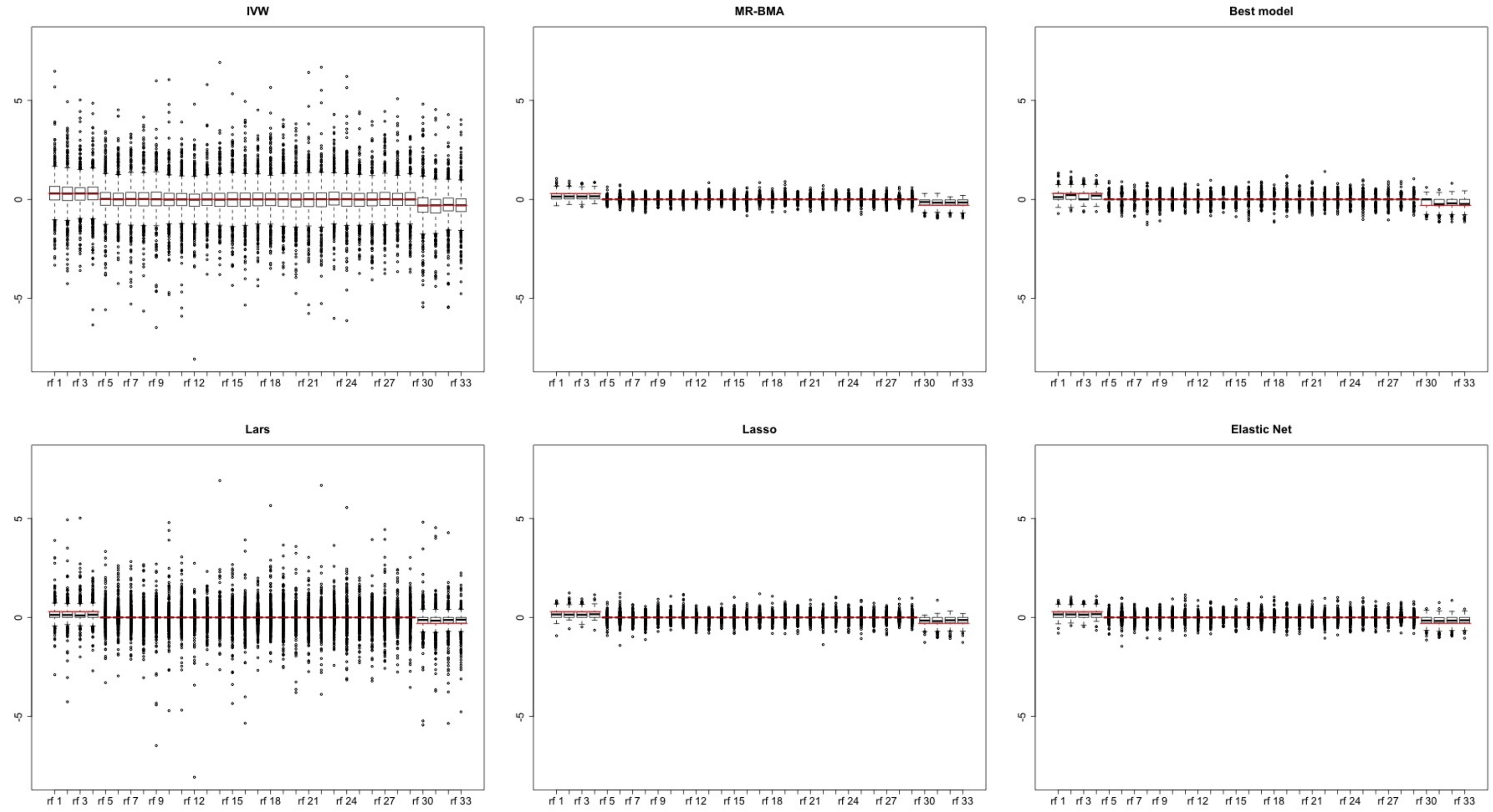

Supplementary Figure 15: Boxplots of the causal effect estimates for setting B for the blood cell traits ($d = 33$), of which the first four have a positive and the last four have a negative causal effect. The true causal effects are marked in red. From top left to bottom right are the competing approaches: IVW, MR-BMA, best model and Lars, Lasso and Elastic Net (all tuned with cross-validation). Proportion of variance explained is set to 0.3.

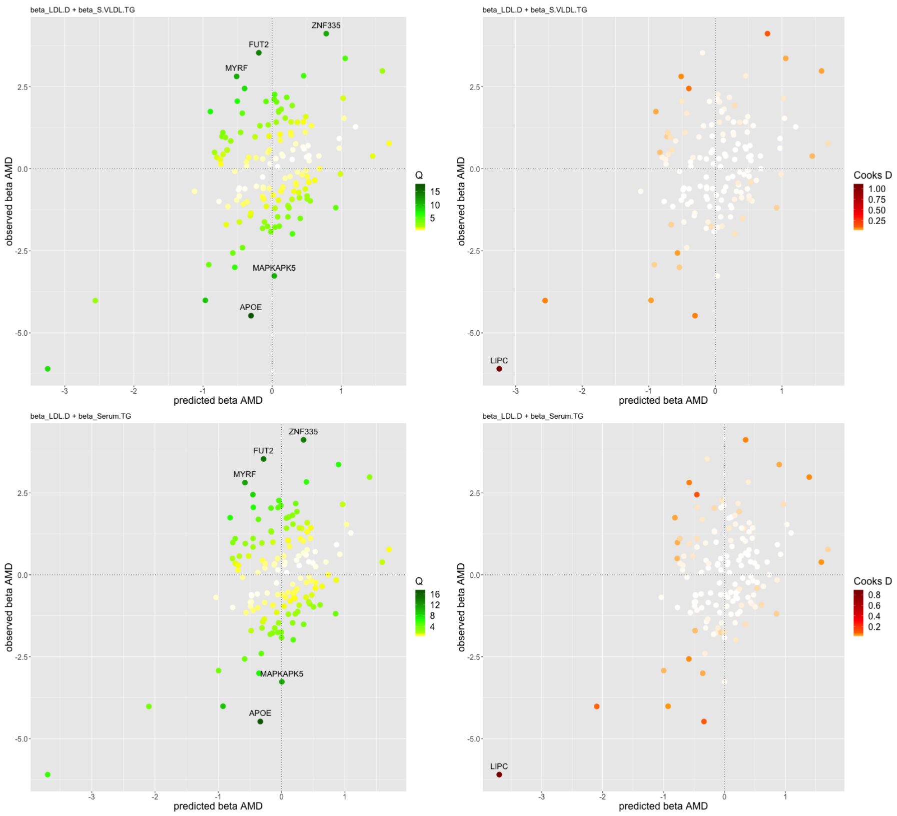

Supplementary Figure 16: Diagnostic plots for all genetic variants ($n = 148$) showing the predicted associations with AMD (x-axis) based on model 2 (M2: LDL diameter (LDL.D) and TG in small VLDL (S.VLDL.TG)), model 3 (M3: LDL.D and Serum.TG), against the observed associations with AMD (y-axis). Model 1 including LDL diameter (LDL.D), and TG content in small HDL (S.HDL.TG) is shown in the main manuscript. The colour code shows: left) the q-statistic for outliers and right) Cook's distance for the influential points. Any genetic variant with q-value larger than 10 or Cook's distance larger than the median is marked by a label indicating the gene region.

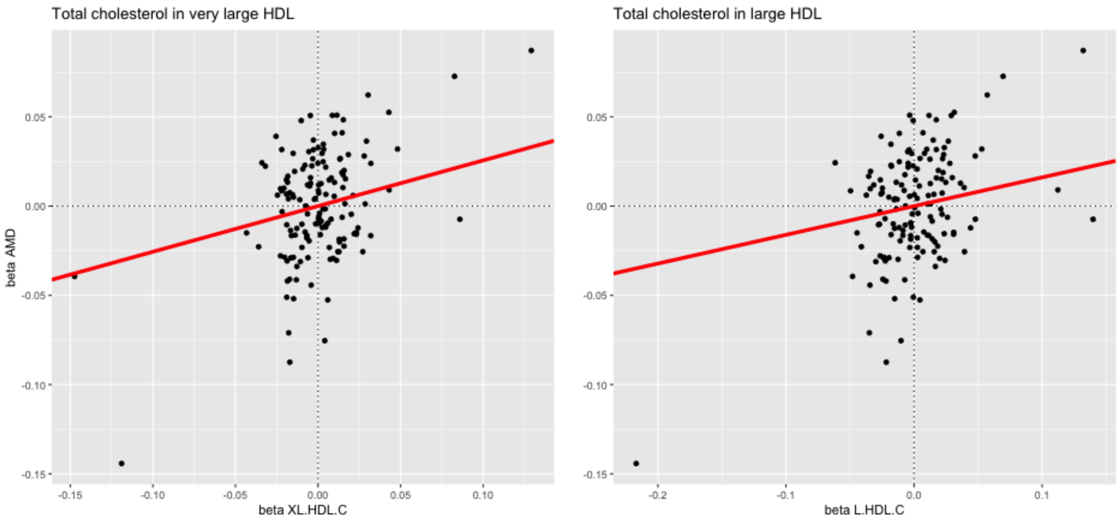

Supplementary Figure 17: Scatterplot for the final set of genetic variants ($n = 145$) plotting the association with A) XL.HDL.C and B) L.HDL.C on the x-axis against the association with AMD on the y-axis after excluding the *LIPC*, *FUT2* and *APOE* gene regions. The model-averaged causal effect (MACE) of each risk factor on AMD is marked in red.

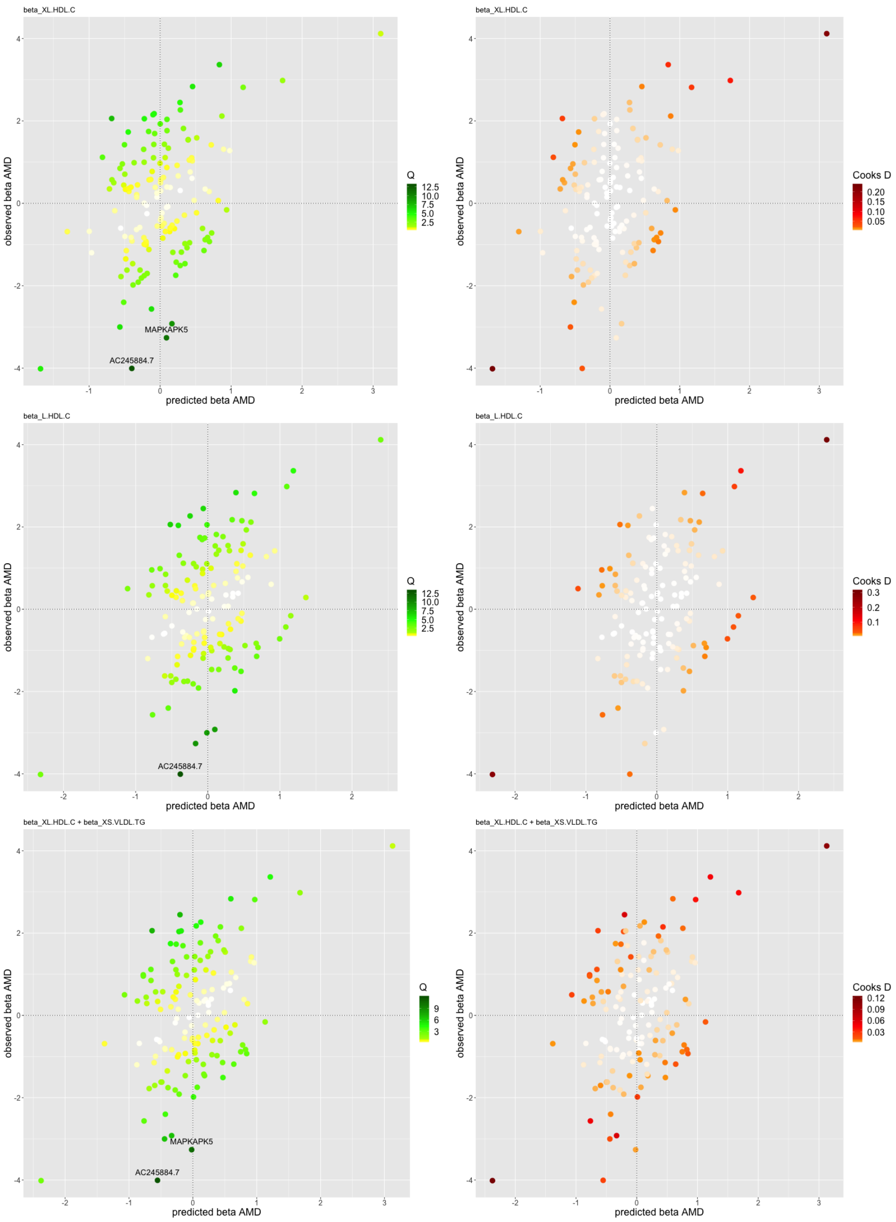

Supplementary Figure 18: Diagnostic plots for the final set of genetic variants ($n = 145$) showing the predicted associations with AMD (x-axis) based on the best individual model 1 (M1: XL.HDL.C), model 2 (M2: L.HDL.C), model 3 (M3: XL.HDL.C and XS.VLDL.TG against the observed associations with AMD (y-axis). The colour code shows: left) the q-statistic for outliers and right) Cook's distance for the influential points. Any genetic variant with q-value larger than 10 or Cook's distance larger than the median is marked by a label indicating the gene region. The *LIPC, FUT2* and *APOE* gene regions have been removed prior to this analysis.

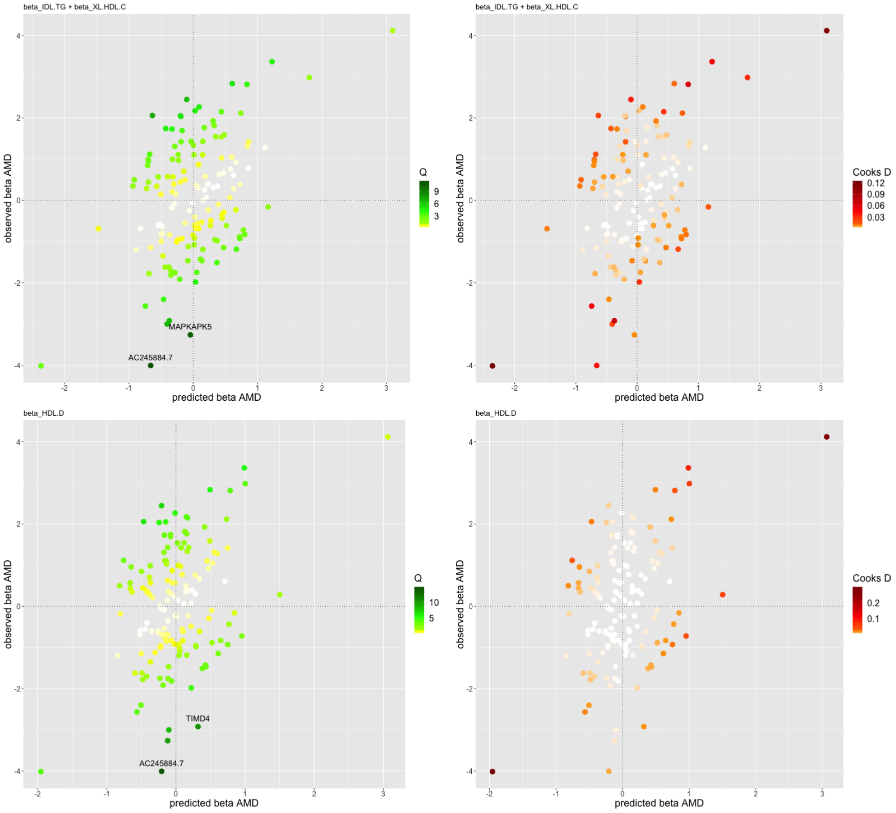

Supplementary Figure 19: Diagnostic plots for the final set of genetic variants ($n = 145$) showing the predicted associations with AMD (x-axis) based on the best individual model 4 (M4: IDL.TG and XL.HDL.C), model 5 (M5: HDL.D) against the observed associations with AMD (y-axis). The colour code shows: left) the q-statistic for outliers and right) Cook's distance for the influential points. Any genetic variant with q-value larger than 10 or Cook's distance larger than the median is marked by a label indicating the gene region. The *LIPC*, *FUT2* and *APOE* gene regions have been removed prior to this analysis.

2 Supplementary Tables

Scenario 1: NMR metabolites, $d = 12$ risk factors															
	Setting A						Setting B								
	$\theta = 0.3$			$\theta = 0$			$\theta = 0.3$			$\theta = -0.3$			$\theta = 0$		
R^2	0.1	0.3	0.5	0.1	0.3	0.5	0.1	0.3	0.5	0.1	0.3	0.5	0.1	0.3	0.5
IVW	0.0014	-0.0099	0.0011	-0.0023	0.0042	-0.0007	-0.0129	0.0012	-0.0015	0.0063	0.0005	-0.0031	0.0072	0.0035	0.0066
Lars	-0.1496	-0.1054	-0.0743	0.0143	0.0179	0.0142	-0.1742	-0.1231	-0.0892	0.1728	0.1230	0.0904	0.0014	-0.0001	-0.0011
Lasso	-0.1337	-0.0864	-0.0628	0.0241	0.0235	0.0195	-0.1678	-0.1030	-0.0730	0.1652	0.1038	0.0706	0.0010	-0.0009	0.0025
Elastic Net	-0.1229	-0.0849	-0.0641	0.0263	0.0247	0.0213	-0.1470	-0.0961	-0.0664	0.1445	0.0962	0.0646	0.0000	0.0009	0.0022
MR-BMA	-0.1263	-0.0889	-0.0625	0.0331	0.0297	0.0240	-0.1739	-0.1468	-0.1309	0.1721	0.1462	0.1301	-0.0006	-0.0012	0.0007
Best model	-0.1225	-0.0724	-0.0483	0.0297	0.0251	0.0189	-0.1663	-0.1282	-0.1181	0.1610	0.1286	0.1205	0.0007	-0.0017	-0.0017

Scenario 2: NMR metabolites, $d = 92$ risk factors															
	Setting A						Setting B								
	$\theta = 0.3$			$\theta = 0$			$\theta = 0.3$			$\theta = -0.3$			$\theta = 0$		
R^2	0.1	0.3	0.5	0.1	0.3	0.5	0.1	0.3	0.5	0.1	0.3	0.5	0.1	0.3	0.5
IVW	-0.1225	0.0181	0.0218	-0.0309	-0.0039	-0.0122	0.0589	-0.0054	0.0023	0.0006	0.0146	0.0199	-0.00191	-0.00087	-0.00015
Lars	-0.2484	-0.2024	-0.1864	0.0038	0.0054	0.0047	-0.2562	-0.2225	-0.2042	0.2576	0.2245	0.2081	0.00045	-0.00014	-0.00012
Lasso	-0.2593	-0.2136	-0.1881	0.0051	0.0070	0.0064	-0.2685	-0.2297	-0.2051	0.2655	0.2297	0.2074	0.00014	-0.00009	-0.00001
Elastic Net	-0.2534	-0.2170	-0.1947	0.0057	0.0072	0.0064	-0.2620	-0.2285	-0.2072	0.2595	0.2291	0.2097	0.00024	-0.00015	-0.00001
MR-BMA	-0.2557	-0.2092	-0.1724	0.0072	0.0078	0.0070	-0.2700	-0.2306	-0.1965	0.2676	0.2307	0.2002	0.00012	0.00010	-0.00012
Best model	-0.2542	-0.2026	-0.1626	0.0070	0.0072	0.0068	-0.2696	-0.2272	-0.1937	0.2634	0.2290	0.1973	0.00030	0.00007	-0.00017

Scenario 3: blood cell traits, $d = 33$ risk factors															
	Setting A						Setting B								
	$\theta = 0.3$			$\theta = 0$			$\theta = 0.3$			$\theta = -0.3$			$\theta = 0$		
R^2	0.1	0.3	0.5	0.1	0.3	0.5	0.1	0.3	0.5	0.1	0.3	0.5	0.1	0.3	0.5
IVW	-0.0027	-0.0161	-0.0082	-0.0015	-0.0021	0.0018	0.0030	-0.0102	-0.0091	-0.0653	-0.0052	-0.0029	0.0186	0.0125	0.0167
Lars	-0.1398	-0.1080	-0.0761	0.0039	0.0042	0.0037	-0.1346	-0.1192	-0.1047	0.1499	0.1173	0.1082	0.0056	0.0125	0.0132
Lasso	-0.1222	-0.0974	-0.0786	0.0058	0.0062	0.0049	-0.1606	-0.1407	-0.1240	0.1670	0.1380	0.1256	0.0112	0.0120	0.0125
Elastic Net	-0.1279	-0.1033	-0.0901	0.0062	0.0070	0.0064	-0.1573	-0.1394	-0.1225	0.1591	0.1386	0.1235	0.0115	0.0118	0.0126
MR-BMA	-0.1057	-0.0637	-0.0427	0.0073	0.0048	0.0029	-0.1623	-0.1332	-0.1370	0.1641	0.1284	0.1393	0.0121	0.0118	0.0121
Best model	-0.0898	-0.0493	-0.0310	0.0064	0.0036	0.0021	-0.1591	-0.1272	-0.0999	0.1615	0.1196	0.1053	0.0126	0.0120	0.0119

Supplementary Table 1: Mean bias of the simulation study (1000 repetitions) for setting A (including four risk factors with positive causal effect $\theta = 0.3$ and zero causal effect otherwise) and setting B (including four risk factors with positive causal effect $\theta = 0.3$ and four risk factor with negative causal effect $\theta = -0.3$ and zero causal effect otherwise). The data scenarios are: Scenario 1: NMR metabolites, $d = 12$ risk factors, Scenario 2: NMR metabolites, $d = 92$ risk factors, Scenario 3: blood cell traits, $d = 33$ risk factors.

Scenario 1: NMR metabolites, $d = 12$ risk factors

	$\theta = 0.3$			$\theta = 0$		
R^2	0.1	0.3	0.5	0.1	0.3	0.5
IVW	0.3014 (0.8469)	0.2901 (0.4177)	0.3011 (0.2898)	-0.0023 (0.8066)	0.0042 (0.4051)	-0.0007 (0.2750)
Lars	0.1504 (0.3855)	0.1946 (0.2353)	0.2257 (0.1914)	0.0143 (0.3289)	0.0179 (0.1829)	0.0142 (0.1531)
Lasso	0.1663 (0.2660)	0.2136 (0.1889)	0.2372 (0.1504)	0.0241 (0.2139)	0.0235 (0.1458)	0.0195 (0.1029)
Elastic Net	0.1771 (0.2752)	0.2151 (0.1753)	0.2359 (0.1407)	0.0263 (0.2343)	0.0247 (0.1593)	0.0213 (0.1091)
MR-BMA	0.1737 (0.2087)	0.2111 (0.1632)	0.2375 (0.1351)	0.0331 (0.1419)	0.0297 (0.0904)	0.0240 (0.0641)
Best model	0.1775 (0.3202)	0.2276 (0.2282)	0.2517 (0.1662)	0.0297 (0.2191)	0.0251 (0.1365)	0.0189 (0.0897)

Scenario 2: NMR metabolites, $d = 92$ risk factors

	$\theta = 0.3$			$\theta = 0$		
R^2	0.1	0.3	0.5	0.1	0.3	0.5
IVW	0.1775 (4.8910)	0.3181 (2.5256)	0.3218 (1.6537)	-0.0309 (4.8750)	-0.0039 (2.4965)	-0.0122 (1.6604)
Lars	0.0516 (0.2159)	0.0976 (0.1936)	0.1136 (0.1576)	0.0038 (0.2026)	0.0054 (0.1484)	0.0047 (0.0848)
Lasso	0.0407 (0.1091)	0.0864 (0.1262)	0.1119 (0.1311)	0.0051 (0.0575)	0.0070 (0.0468)	0.0064 (0.0402)
Elastic Net	0.0466 (0.1071)	0.0830 (0.1094)	0.1053 (0.1110)	0.0057 (0.0658)	0.0072 (0.0435)	0.0064 (0.0335)
MR-BMA	0.0443 (0.1107)	0.0908 (0.1345)	0.1276 (0.1420)	0.0072 (0.0436)	0.0078 (0.0369)	0.0070 (0.0325)
Best model	0.0458 (0.1887)	0.0974 (0.1977)	0.1374 (0.1951)	0.0070 (0.0899)	0.0072 (0.0737)	0.0068 (0.0629)

Scenario 3: blood cell traits, $d = 33$ risk factors

	$\theta = 0.3$			$\theta = 0$		
R^2	0.1	0.3	0.5	0.1	0.3	0.5
IVW	0.2973 (1.3937)	0.2839 (0.6799)	0.2918 (0.4254)	-0.0015 (1.2552)	-0.0021 (0.6499)	0.0018 (0.4162)
Lars	0.1602 (0.6386)	0.1920 (0.3588)	0.2239 (0.2404)	0.0039 (0.5788)	0.0042 (0.3341)	0.0037 (0.2146)
Lasso	0.1778 (0.1826)	0.2026 (0.1336)	0.2214 (0.1156)	0.0058 (0.1080)	0.0062 (0.0613)	0.0049 (0.0429)
Elastic Net	0.1721 (0.1672)	0.1967 (0.1250)	0.2099 (0.1063)	0.0062 (0.1136)	0.0070 (0.0690)	0.0064 (0.0477)
MR-BMA	0.1943 (0.1463)	0.2363 (0.1187)	0.2573 (0.0956)	0.0073 (0.0545)	0.0048 (0.0366)	0.0029 (0.0263)
Best model	0.2102 (0.2163)	0.2507 (0.1521)	0.2690 (0.1146)	0.0064 (0.0835)	0.0036 (0.0471)	0.0021 (0.0335)

Supplementary Table 2: Mean and standard deviation (in round brackets) of the causal effect estimate from the simulation study (1000 repetitions) for Setting A (including four risk factors with positive causal effect $\theta = 0.3$ and zero causal effect otherwise). The data scenarios are: Scenario 1: NMR metabolites, $d = 12$ risk factors, Scenario 2: NMR metabolites, $d = 92$ risk factors, Scenario 3: blood cell traits, $d = 33$ risk factors.

Scenario 1: NMR metabolites, $d = 12$ risk factors

	$\theta = 0.3$			$\theta = -0.3$			$\theta = 0$		
R^2	0.1	0.3	0.5	0.1	0.3	0.5	0.1	0.3	0.5
IVW	0.2871 (0.777)	0.3012 (0.384)	0.2985 (0.250)	-0.2937 (0.752)	-0.2995 (0.406)	-0.3031 (0.245)	0.0072 (0.785)	0.0035 (0.417)	0.0066 (0.258)
Lars	0.1258 (0.380)	0.1769 (0.238)	0.2108 (0.196)	-0.1272 (0.365)	-0.1770 (0.251)	-0.2096 (0.185)	0.0014 (0.361)	-0.0001 (0.210)	-0.0011 (0.151)
Lasso	0.1322 (0.336)	0.1970 (0.220)	0.2270 (0.176)	-0.1348 (0.274)	-0.1962 (0.215)	-0.2294 (0.177)	0.0010 (0.266)	-0.0009 (0.187)	0.0025 (0.140)
Elastic Net	0.1530 (0.354)	0.2039 (0.212)	0.2336 (0.171)	-0.1555 (0.296)	-0.2038 (0.208)	-0.2354 (0.168)	0.0000 (0.305)	0.0009 (0.193)	0.0022 (0.142)
MR-BMA	0.1261 (0.198)	0.1532 (0.170)	0.1691 (0.160)	-0.1279 (0.199)	-0.1538 (0.170)	-0.1699 (0.162)	-0.0006 (0.146)	-0.0012 (0.098)	0.0007 (0.079)
Best model	0.1337 (0.297)	0.1718 (0.225)	0.1819 (0.191)	-0.1390 (0.297)	-0.1714 (0.225)	-0.1795 (0.190)	0.0007 (0.215)	-0.0017 (0.143)	-0.0017 (0.107)

Scenario 2: NMR metabolites, $d = 92$ risk factors

	$\theta = 0.3$			$\theta = -0.3$			$\theta = 0$		
R^2	0.1	0.3	0.5	0.1	0.3	0.5	0.1	0.3	0.5
IVW	0.3589 (4.858)	0.2946 (2.534)	0.3023 (1.545)	-0.2994 (4.512)	-0.2854 (2.358)	-0.2801 (1.609)	-0.0019 (4.834)	-0.0009 (2.398)	-0.0002 (1.575)
Lars	0.0438 (0.171)	0.0775 (0.169)	0.0958 (0.152)	-0.0424 (0.171)	-0.0755 (0.169)	-0.0919 (0.149)	0.0005 (0.161)	-0.0001 (0.126)	-0.0001 (0.097)
Lasso	0.0315 (0.107)	0.0703 (0.122)	0.0949 (0.130)	-0.0345 (0.108)	-0.0703 (0.123)	-0.0926 (0.128)	0.0001 (0.060)	-0.0001 (0.054)	0.0000 (0.049)
Elastic Net	0.0380 (0.098)	0.0715 (0.111)	0.0928 (0.116)	-0.0405 (0.105)	-0.0709 (0.110)	-0.0903 (0.113)	0.0002 (0.058)	-0.0002 (0.049)	0.0000 (0.045)
MR-BMA	0.0300 (0.097)	0.0694 (0.127)	0.1035 (0.139)	-0.0324 (0.099)	-0.0693 (0.126)	-0.0998 (0.136)	0.0001 (0.043)	0.0001 (0.041)	-0.0001 (0.037)
Best model	0.0304 (0.150)	0.0728 (0.174)	0.1063 (0.173)	-0.0366 (0.170)	-0.0710 (0.172)	-0.1027 (0.172)	0.0003 (0.085)	0.0001 (0.074)	-0.0002 (0.063)

Scenario 3: blood cell traits, $d = 33$ risk factors

	$\theta = 0.3$			$\theta = -0.3$			$\theta = 0$		
R^2	0.1	0.3	0.5	0.1	0.3	0.5	0.1	0.3	0.5
IVW	0.3030 (1.463)	0.2898 (0.779)	0.2909 (0.479)	-0.3653 (1.527)	-0.3052 (0.810)	-0.3029 (0.508)	0.0066 (1.528)	0.0005 (0.783)	0.0047 (0.507)
Lars	0.1654 (0.762)	0.1808 (0.379)	0.1953 (0.297)	-0.1501 (0.766)	-0.1827 (0.461)	-0.1918 (0.304)	-0.0064 (0.762)	0.0005 (0.394)	0.0012 (0.274)
Lasso	0.1394 (0.206)	0.1593 (0.164)	0.1760 (0.149)	-0.1330 (0.188)	-0.1620 (0.167)	-0.1744 (0.149)	-0.0008 (0.159)	0.0000 (0.105)	0.0005 (0.078)
Elastic Net	0.1427 (0.218)	0.1606 (0.156)	0.1775 (0.139)	-0.1409 (0.206)	-0.1614 (0.158)	-0.1765 (0.143)	-0.0005 (0.184)	-0.0002 (0.109)	0.0006 (0.087)
MR-BMA	0.1377 (0.177)	0.1668 (0.150)	0.1630 (0.151)	-0.1359 (0.173)	-0.1716 (0.155)	-0.1607 (0.149)	0.0001 (0.096)	-0.0002 (0.072)	0.0001 (0.053)
Best model	0.1409 (0.266)	0.1728 (0.203)	0.2001 (0.180)	-0.1385 (0.252)	-0.1804 (0.204)	-0.1947 (0.177)	0.0006 (0.160)	0.0000 (0.102)	-0.0001 (0.078)

Supplementary Table 3: Mean and standard deviation (in round brackets) of the causal effect estimate from the simulation study (1000 repetitions) for Setting B (including four risk factors with positive causal effect $\theta = 0.3$ and four risk factor with negative causal effect $\theta = -0.3$ and zero causal effect otherwise). The data scenarios are: Scenario 1: NMR metabolites, $d = 12$ risk factors, Scenario 2: NMR metabolites, $d = 92$ risk factors, Scenario 3: blood cell traits, $d = 33$ risk factors.

A) Model averaging

	risk factor	*MIP*	$\hat{\theta}_{\text{MACE}}$
1	LDL.D	0.527	-0.229
2	XS.VLDL.TG	0.247	-0.124
3	S.HDL.TG	0.236	-0.101
4	IDL.TG	0.213	-0.108
5	XXL.VLDL.TG	0.188	0.095
6	S.VLDL.TG	0.175	-0.070
7	S.LDL.C	0.137	0.059
8	Serum.TG	0.137	-0.062
9	Est.C	0.097	0.030
10	XL.HDL.C	0.085	0.021

B) Individual models

	risk factor(s)	*PP*	$\hat{\theta}_{\gamma}$
1	LDL.D,S.HDL.TG	0.062	-0.376,-0.398
2	LDL.D,S.VLDL.TG	0.052	-0.485,-0.379
3	LDL.D,Serum.TG	0.020	-0.454,-0.365
4	S.HDL.TG	0.019	-0.433
5	Est.C,IDL.TG	0.019	0.393,-0.625
6	LDL.D,XS.VLDL.TG	0.018	-0.339,-0.324
7	XS.VLDL.TG	0.017	-0.373
8	LDL.D,M.VLDL.TG	0.014	-0.545,-0.408
9	S.HDL.TG,XXL.VLDL.TG	0.013	-0.653,0.45
10	IDL.TG	0.009	-0.343

Supplementary Table 4: Ranking of risk factors (top ten) for age-related macular degeneration including all variants

Ranking of risk factors (top ten) for age-related macular degeneration according to their marginal inclusion probability (*MIP*) A) and the best ten individual models (sets of risk factors) according to their posterior probability (*PP*) B). Calculation is based on all genetic variants $n = 148$ including the *LIPC* region. $\hat{\theta}_{\gamma}$ is the causal effect estimate for a specific model and $\hat{\theta}_{\text{MACE}}$ is the model averaged causal effect of a risk factor.

	rs	region	q M1	q M2	q M3	max q
1	rs6859	APOE	17.007	17.388	17.132	17.388
2	rs492602	FUT2	15.526	13.899	14.591	15.526
3	rs4465830	ZNF335	7.395	11.127	14.223	14.223
4	rs174532	MYRF	11.939	11.078	11.517	11.939
5	rs6489818	MAPKAPK5	11.226	10.857	10.68	11.226
6	rs103294	AC245884.7	8.857	9.255	9.504	9.504
7	rs3817588	GCKR	7.263	8.095	8.411	8.411
8	rs261342	LIPC	7.11	8.107	5.747	8.107
9	rs903319	SLC2A2	8.06	6.567	6.276	8.06
10	rs2587534	AL160408.6	6.498	6.063	6.999	6.999
11	rs2710642	EHBP1	6.662	6.955	6.538	6.955
12	rs9491696	RSPO3	6.317	5.658	5.966	6.317
13	rs1689797	LINC01344	4.638	5.325	6.079	6.079
14	rs6882076	TIMD4	5.742	4.023	3.706	5.742
15	rs8176720	ABO	5.415	4.972	5.334	5.415
16	rs688	LDLR	4.85	5.178	4.694	5.178
17	rs1781930	AKR1C8P	4.978	4.585	4.445	4.978
18	rs702485	DAGLB	4.863	3.892	4.335	4.863
19	rs38855	MET	4.636	3.896	4.858	4.858
20	rs2925979	CMIP	4.66	4.516	4.243	4.66
21	rs7703051	HMGCR	4.581	3.988	3.928	4.581
22	rs2602836	ADH5	3.724	4.357	4.528	4.528
23	rs3741414	INHBC	3.873	4.434	4.158	4.434
24	rs4148218	ABCG8	3.967	3.592	3.666	3.967
25	rs3822072	FAM13A	3.549	3.858	3.811	3.858
26	rs5880	CETP	1.127	2.123	3.679	3.679
27	rs6680658	GALNT2	3.124	3.675	3.457	3.675
28	rs9930333	FTO	3.351	3.428	3.04	3.428
29	rs7225700	THCAT158	3.127	3.305	3.381	3.381
30	rs217386	NPC1L1	1.959	3.311	2.665	3.311

Supplementary Table 5: This table displays the 30 variants with the largest maximum q and the region they fall in based on all $n = 148$ genetic variants for the best individual model 1 (M1: LDL.D and S.HDL.TG), model 2 (M2: LDL.D and S.VLDL.TG), and model 3 (M3: LDL.D and Serum.TG) and the maximum q of each variant in all models used for diagnostics.

	rs	region	Cd M1	Cd M2	Cd M3	max Cd
1	rs261342	LIPC	**0.989**	**1.087**	**0.871**	1.087
2	rs4465830	ZNF335	0.188	0.108	0.056	0.188
3	rs3817588	GCKR	0.058	0.085	0.105	0.105
4	rs6859	APOE	0.081	0.076	0.087	0.087
5	rs5880	CETP	0.056	0.071	0.081	0.081
6	rs174532	MYRF	0.062	0.062	0.061	0.062
7	rs686030	TTC39B	0.054	0.04	0.052	0.054
8	rs7703051	HMGCR	0.039	0.045	0.05	0.05
9	rs103294	AC245884.7	0.045	0.044	0.044	0.045
10	rs10401969	SUGP1	0.009	0.025	0.043	0.043
11	rs1689797	LINC01344	0.037	0.031	0.026	0.037
12	rs2710642	EHBP1	0.031	0.033	0.03	0.033
13	rs2587534	AL160408.6	0.02	0.018	0.024	0.024
14	rs10493326	DOCK7	0.011	0.017	0.023	0.023
15	rs894210	intergenic	0.015	0.022	0.02	0.022
16	rs6882076	TIMD4	0.006	0.016	0.02	0.02
17	rs903319	SLC2A2	0.02	0.008	0.007	0.02
18	rs515135	APOB(intergenic)	0.019	0.011	0.012	0.019
19	rs799160	intergenic	0.017	0.016	0.019	0.019
20	rs3741414	INHBC	0.01	0.016	0.013	0.016
21	rs1515110	NR	0.014	0.01	0.007	0.014
22	rs1800562	HFE	0.01	0.012	0.012	0.012
23	rs2068888	CYP26A1	0.012	0.011	0.011	0.012
24	rs7225700	THCAT158	0.011	0.012	0.012	0.012
25	rs492602	FUT2	0.011	0.002	0.005	0.011
26	rs38855	MET	0.008	0.003	0.01	0.01
27	rs688	LDLR	0.007	0.01	0.006	0.01
28	rs6680658	GALNT2	0.005	0.01	0.009	0.01
29	rs3198697	PDXDC1	0.007	0.01	0.01	0.01
30	rs2326077	intergenic	0.006	0.006	0.01	0.01
	threshold		0.696	0.696	0.696	

Supplementary Table 6: This table displays the 30 variants with the largest maximum Cook's distance (Cd) and the region they fall based on all $n = 148$ genetic variants including *LIPC* for the best individual model 1 (M1: LDL.D and S.HDL.TG), model 2 (M2: LDL.D and S.VLDL.TG), and model 3 (M3: LDL.D and Serum.TG). The final line gives the suggested cut-off for Cook's distance and variants with Cd above this threshold are given in bold.

	risk factor	*MIP*	$\hat{\theta}_{\text{MACE}}$
1	XL.HDL.C	0.700	0.344
2	L.HDL.C	0.229	0.087
3	HDL.D	0.087	0.022
4	XS.VLDL.TG	0.082	-0.019
5	LDL.D	0.074	-0.018
6	IDL.TG	0.066	-0.012
7	XXL.VLDL.TG	0.063	0.018
8	S.VLDL.TG	0.062	-0.014
9	Serum.TG	0.061	-0.014
10	Serum.C	0.054	-0.011
11	HDL.C	0.051	0.009
12	M.HDL.C	0.048	-0.010
13	S.HDL.TG	0.047	-0.006
14	XL.HDL.TG	0.045	0.005
15	M.VLDL.C	0.043	-0.005
16	S.VLDL.C	0.043	-0.005
17	ApoA1	0.040	-0.007
18	M.VLDL.TG	0.039	0.006
19	ApoB	0.038	-0.004
20	L.VLDL.C	0.038	-0.005
21	XL.VLDL.TG	0.034	-0.003
22	L.VLDL.TG	0.033	-0.001
23	S.LDL.C	0.033	0.001
24	LDL.C	0.031	-0.003
25	IDL.C	0.029	-0.001
26	SM	0.027	-0.003
27	VLDL.D	0.027	0.002
28	Tot.FA	0.026	-0.001
29	Est.C	0.026	0.001
30	TotPG	0.026	-0.002

Supplementary Table 7: Ranking of risk factors for age-related macular degeneration according to their marginal inclusion probability (*MIP*) after excluding genetic variants in the *LIPC, FUT2* and *APOE* region (n = 145). Abbreviations: *MIP*=marginal inclusion probability, MACE=model-averaged causal effect.

	rs	region	Q M1	Q M2	Q M3	Q M4	Q M5	max Q
1	rs103294	AC245884.7	13.03	13.155	11.936	11.203	14.449	14.449
2	rs6489818	MAPKAPK5	11.244	9.575	10.53	10.356	9.883	11.244
3	rs6882076	TIMD4	9.536	9.118	6.708	6.503	10.504	10.504
4	rs2587534	AL160408.6	5.931	8.936	6.551	6.735	8.409	8.936
5	rs903319	SLC2A2	7.514	6.651	7.275	7.255	6.379	7.514
6	rs3817588	GCKR	4.698	6.3	7.015	6.495	7.051	7.051
7	rs1689797	LINC01344	6.403	4.747	4.635	4.587	5.648	6.403
8	rs8176720	ABO	3.929	6.312	4.592	4.734	5.197	6.312
9	rs38855	MET	3.768	5.98	5.082	4.973	5.205	5.98
10	rs9491696	RSPO3	5.651	5.974	5.017	4.971	5.479	5.974
11	rs7703051	HMGCR	5.974	3.24	3.246	3.319	4.009	5.974
12	rs688	LDLR	2.562	5.557	3.97	4.071	4.856	5.557
13	rs5880	CETP	5.433	2.877	2.687	2.73	4.246	5.433
14	rs1781930	AKR1C8P	5.176	4.259	4.996	5.072	4.851	5.176
15	rs3822072	FAM13A	5.105	3.376	4.504	4.606	4.099	5.105
16	rs2923084	AMPD3	5.067	2.814	2.956	2.944	3.933	5.067
17	rs9693857	AC022784.6	4.752	3.147	3.966	4.246	3.601	4.752
18	rs2710642	EHBP1	3.632	3.432	4.381	4.714	3.318	4.714
19	rs174532	MYRF	2.708	4.701	3.405	3.927	4.12	4.701
20	rs6680658	GALNT2	3.216	3.885	3.926	3.527	3.577	3.926
21	rs686030	TTC39B	1.58	3.558	1.7	1.393	3.913	3.913
22	rs702485	DAGLB	3.569	3.439	3.887	3.768	3.597	3.887
23	rs9930333	FTO	3.872	2.154	3.299	3.245	2.299	3.872
24	rs17789218	intergenic	3.72	2.12	3.145	3.219	3.512	3.72
25	rs2068888	CYP26A1	3.714	1.944	2.47	2.627	2.291	3.714
26	rs9686661	C5orf67	3.702	1.258	2.31	2.597	1.811	3.702
27	rs2297374	SLC22A1	3.294	2.614	2.716	2.554	3.608	3.608
28	rs2925979	CMIP	3.135	3.14	3.417	3.486	3.142	3.486
29	rs3741414	INHBC	2.203	2.149	3.335	3.438	1.8	3.438
30	rs7264396	FER1L4	2.74	3.251	2.562	2.372	3.438	3.438

Supplementary Table 8: This table displays the 30 variants with the largest maximum q-statistic and the region they fall in based on $n = 145$ genetic variants after excluding *LIPC, FUT2* and *APOE* for the best individual model 1 (M1: XL.HDL.C), model 2 (M2: L.HDL.C), model 3 (M3: XL.HDL.C and XS.VLDL.TG), model 4 (M4: IDL.TG and XL.HDL.C), model 5 (M5: HDL.D), and the maximum q-statistic of each variant in all models used for diagnostics.

	rs	region	Cd M1	Cd M2	Cd M3	Cd M4	Cd M5	max Cd
1	rs4465830	ZNF335	0.216	0.311	0.106	0.113	0.271	0.311
2	rs5880	CETP	0.234	0.277	0.122	0.122	0.297	0.297
3	rs1689797	LINC01344	0.061	0.098	0.047	0.048	0.086	0.098
4	rs686030	TTC39B	0.072	0.062	0.04	0.033	0.062	0.072
5	rs6882076	TIMD4	0.004	0.001	0.061	0.07	0.016	0.07
6	rs3817588	GCKR	0.005	0	0.062	0.037	0.005	0.062
7	rs174532	MYRF	0.052	0.027	0.039	0.057	0.039	0.057
8	rs13107325	SLC39A8	0.001	0.032	0.001	0	0.056	0.056
9	rs7703051	HMGCR	0.001	0.027	0.05	0.048	0.019	0.05
10	rs903319	SLC2A2	0.047	0.025	0.024	0.024	0.021	0.047
11	rs894210	intergenic	0.008	0.046	0.023	0.011	0.018	0.046
12	rs10773105	SCARB1	0.015	0.042	0.009	0.008	0.04	0.042
13	rs103294	AC245884.7	0.028	0.026	0.023	0.039	0.009	0.039
14	rs998584	VEGFA(intergenic)	0	0.039	0.005	0.002	0.013	0.039
15	rs17789218	intergenic	0.034	0.003	0.017	0.017	0.031	0.034
16	rs1800961	HNF4A	0.015	0.033	0.013	0.014	0.011	0.033
17	rs2923084	AMPD3	0.001	0.009	0.03	0.031	0.002	0.031
18	rs688	LDLR	0.005	0.011	0.025	0.029	0.004	0.029
19	rs2587534	AL160408.6	0.026	0	0.018	0.021	0.001	0.026
20	rs1515110	NR	0.008	0.025	0.012	0.008	0.016	0.025
21	rs7897379	REEP3	0.017	0.018	0.013	0.009	0.024	0.024
22	rs10493326	DOCK7	0.003	0.017	0.021	0.014	0.004	0.021
23	rs499974	RN7SL786P	0.016	0.021	0.009	0.009	0.018	0.021
24	rs9491696	RSPO3	0.016	0.013	0.01	0.011	0.02	0.02
25	rs3741414	INHBC	0.003	0.002	0.016	0.019	0.001	0.019
26	rs9686661	C5orf67	0.013	0.002	0.017	0.014	0	0.017
27	rs38855	MET	0	0.014	0.017	0.015	0.005	0.017
28	rs2602836	ADH5	0.016	0.011	0.011	0.011	0.016	0.016
29	rs2278236	ANGPTL4	0.01	0.015	0.005	0.005	0.01	0.015
30	rs702485	DAGLB	0.013	0.014	0.008	0.007	0.014	0.014
			0.457	0.457	0.697	0.697	0.457	

Supplementary Table 9: This table displays the 30 variants with the largest maximum Cook's distance (Cd) and the region they fall in based on $n = 145$ genetic variants after excluding *LIPC, FUT2* and *APOE* for the best individual model 1 (M1: XL.HDL.C), model 2 (M2: L.HDL.C), model 3 (M3: XL.HDL.C and XS.VLDL.TG), model 4 (M4: IDL.TG and XL.HDL.C), model 5 (M5: HDL.D), the final line gives the suggested cut-off for Cook's distance and this time, there are no variants with Cd above this threshold.

$p = 0.01$

#	risk factor	*MIP*	$\hat{\theta}_{\text{MACE}}$
1	XL.HDL.C	0.608	0.308
2	L.HDL.C	0.283	0.109
3	HDL.D	0.087	0.030
4	HDL.C	0.024	0.008
5	XS.VLDL.TG	0.011	-0.002
6	IDL.TG	0.009	-0.002
7	S.HDL.TG	0.009	-0.002
8	LDL.D	0.007	-0.002
9	Serum.C	0.007	-0.001
10	S.VLDL.TG	0.007	-0.001

$p = 0.05$

#	risk factor	*MIP*	$\hat{\theta}_{\text{MACE}}$
1	XL.HDL.C	0.663	0.330
2	L.HDL.C	0.249	0.095
3	HDL.D	0.084	0.026
4	XS.VLDL.TG	0.047	-0.010
5	IDL.TG	0.040	-0.007
6	LDL.D	0.037	-0.008
7	HDL.C	0.035	0.008
8	S.VLDL.TG	0.032	-0.006
9	Serum.C	0.030	-0.005
10	Serum.TG	0.029	-0.006

$p = 0.1$

#	risk factor	*MIP*	$\hat{\theta}_{\text{MACE}}$
1	XL.HDL.C	0.70	0.34
2	L.HDL.C	0.23	0.09
3	HDL.D	0.09	0.02
4	XS.VLDL.TG	0.08	-0.02
5	LDL.D	0.07	-0.02
6	IDL.TG	0.07	-0.01
7	S.VLDL.TG	0.06	-0.01
8	XXL.VLDL.TG	0.06	0.02
9	Serum.TG	0.06	-0.01
10	Serum.C	0.05	-0.01

$p = 0.2$

#	risk factor	*MIP*	$\hat{\theta}_{\text{MACE}}$
1	XL.HDL.C	0.700	0.344
2	L.HDL.C	0.229	0.087
3	HDL.D	0.087	0.022
4	XS.VLDL.TG	0.082	-0.019
5	LDL.D	0.075	-0.018
6	IDL.TG	0.067	-0.013
7	S.VLDL.TG	0.062	-0.014
8	XXL.VLDL.TG	0.061	0.018
9	Serum.TG	0.061	-0.014
10	Serum.C	0.053	-0.010

$p = 0.3$

#	risk factor	*MIP*	$\hat{\theta}_{\text{MACE}}$
1	XL.HDL.C	0.675	0.315
2	L.HDL.C	0.302	0.126
3	XXL.VLDL.TG	0.300	0.121
4	LDL.D	0.244	-0.073
5	Serum.TG	0.212	-0.065
6	XS.VLDL.TG	0.197	-0.052
7	S.VLDL.TG	0.190	-0.053
8	M.VLDL.TG	0.173	0.048
9	Serum.C	0.165	-0.053
10	ApoA1	0.152	-0.038

Supplementary Table 10: Parameter check for the prior probability p, ranging from $p = 0.01$ to $p = 0.3$. This reflects 0.3 to 9 expected causal risk factors. The main analysis used $p = 0.1$ reflecting an a priori expected number of 3 causal risk factors. Abbreviations: *MIP*=marginal inclusion probability, MACE=model-averaged causal effect.

$\sigma = 0.1$

#	risk factor	*MIP*	$\hat{\theta}_{\text{MACE}}$
1	XL.HDL.C	0.52	0.13
2	L.HDL.C	0.42	0.09
3	HDL.D	0.27	0.05
4	LDL.D	0.15	-0.02
5	HDL.C	0.14	0.02
6	XS.VLDL.TG	0.13	-0.02
7	S.HDL.TG	0.13	-0.02
8	S.VLDL.TG	0.11	-0.01
9	IDL.TG	0.10	-0.01
10	Serum.TG	0.09	-0.01

$\sigma = 0.3$

#	risk factor	*MIP*	$\hat{\theta}_{\text{MACE}}$
1	XL.HDL.C	0.69	0.32
2	L.HDL.C	0.25	0.09
3	XS.VLDL.TG	0.11	-0.02
4	HDL.D	0.11	0.03
5	LDL.D	0.10	-0.02
6	IDL.TG	0.08	-0.01
7	S.VLDL.TG	0.08	-0.02
8	XXL.VLDL.TG	0.08	0.02
9	Serum.TG	0.07	-0.01
10	S.HDL.TG	0.06	-0.01

$\sigma = 0.5$

#	risk factor	*MIP*	$\hat{\theta}_{\text{MACE}}$
1	XL.HDL.C	0.70	0.34
2	L.HDL.C	0.23	0.09
3	HDL.D	0.09	0.02
4	XS.VLDL.TG	0.08	-0.02
5	LDL.D	0.07	-0.02
6	IDL.TG	0.07	-0.01
7	S.VLDL.TG	0.06	-0.01
8	XXL.VLDL.TG	0.06	0.02
9	Serum.TG	0.06	-0.01
10	Serum.C	0.05	-0.01

$\sigma = 0.7$

#	risk factor	*MIP*	$\hat{\theta}_{\text{MACE}}$
1	XL.HDL.C	0.69	0.35
2	L.HDL.C	0.23	0.09
3	HDL.D	0.08	0.02
4	XS.VLDL.TG	0.07	-0.02
5	LDL.D	0.06	-0.01
6	IDL.TG	0.05	-0.01
7	S.VLDL.TG	0.05	-0.01
8	Serum.TG	0.05	-0.01
9	XXL.VLDL.TG	0.05	0.02
10	Serum.C	0.05	-0.01

Supplementary Table 11: Parameter check for the prior variance σ^2, ranging from $\sigma = 0.1$ to $\sigma = 0.7$. The main analysis used $\sigma = 0.5$. Abbreviations: *MIP*=marginal inclusion probability, MACE=model-averaged causal effect.

	risk factor	beta	$p-$value
1	Serum.C	-2.033	0.004
2	LDL.C	-1.808	0.014
3	IDL.C	2.156	0.014
4	XXL.VLDL.TG	1.075	0.015
5	M.VLDL.TG	1.769	0.019
6	LDL.D	-0.937	0.032
7	S.LDL.C	1.302	0.064
8	S.VLDL.C	1.046	0.066
9	L.HDL.C	1.350	0.129
10	S.HDL.TG	0.562	0.175
11	SM	-0.221	0.223
12	VLDL.D	-0.497	0.250
13	ApoA1	-0.390	0.318
14	XS.VLDL.TG	-1.015	0.330
15	M.VLDL.C	-0.856	0.339
16	Tot.FA	0.350	0.359
17	L.VLDL.TG	-0.616	0.371
18	TotPG	-0.246	0.470
19	Serum.TG	-0.771	0.525
20	XL.VLDL.TG	-0.302	0.605
21	IDL.TG	0.398	0.654
22	ApoB	0.273	0.658
23	L.VLDL.C	-0.241	0.670
24	M.HDL.C	0.098	0.814
25	Est.C	0.082	0.828
26	HDL.C	-0.193	0.838
27	XL.HDL.TG	0.083	0.850
28	XL.HDL.C	0.079	0.868
29	S.VLDL.TG	0.066	0.932
30	HDL.D	-0.029	0.958

Supplementary Table 12: Ranking of risk factors for age-related macular degeneration using inverse-variance weighted (IVW) regression according to their $p-$value after excluding genetic variants in the *LIPC, FUT2* and *APOE* region $n = 145$. Abbreviations: beta=causal effect, p=p-value of the causal effect (not adjusted for multiple testing).

	risk factor	beta L1
1	L.HDL.C	0.357
2	LDL.D	-0.255
3	XXL.VLDL.TG	0.251
4	S.VLDL.TG	-0.170
5	M.HDL.C	-0.157
6	XL.HDL.C	0.115
7	XL.VLDL.TG	-0.104
8	ApoA1	-0.093
9	Est.C	0.062
10	Serum.TG	-0.010
11	SM	-0.005
	ApoB	0
	HDL.C	0
	HDL.D	0
	IDL.C	0
	IDL.TG	0
	L.VLDL.C	0
	L.VLDL.TG	0
	LDL.C	0
	M.VLDL.C	0
	M.VLDL.TG	0
	S.HDL.TG	0
	S.LDL.C	0
	S.VLDL.C	0
	Serum.C	0
	Tot.FA	0
	TotPG	0
	VLDL.D	0
	XL.HDL.TG	0
	XS.VLDL.TG	0

Supplementary Table 13: Ranking of risk factors for age-related macular degeneration using Lars regression according to their L1 regularised causal effect estimate after excluding genetic variants in the *LIPC, FUT2* and *APOE* region $n = 145$. Abbreviations: beta L1=L1 regularised causal effect.

	risk factor	beta L1
1	XL.HDL.C	0.306
2	XS.VLDL.TG	-0.102
3	L.HDL.C	0.092
4	LDL.D	-0.039
	ApoA1	0
	ApoB	0
	Est.C	0
	HDL.C	0
	HDL.D	0
	IDL.C	0
	IDL.TG	0
	L.VLDL.C	0
	L.VLDL.TG	0
	LDL.C	0
	M.HDL.C	0
	M.VLDL.C	0
	M.VLDL.TG	0
	S.HDL.TG	0
	S.LDL.C	0
	S.VLDL.C	0
	S.VLDL.TG	0
	Serum.C	0
	Serum.TG	0
	SM	0
	Tot.FA	0
	TotPG	0
	VLDL.D	0
	XL.HDL.TG	0
	XL.VLDL.TG	0
	XXL.VLDL.TG	0

Supplementary Table 14: Ranking of risk factors for age-related macular degeneration using Lasso regression (L1 penalty) according to their regularised causal effect estimate after excluding genetic variants in the *LIPC, FUT2* and *APOE* region $n = 145$. Abbreviations: beta L1=L1 regularised causal effect.

	risk factor	beta L1+L2
1	L.HDL.C	0.269
2	XL.HDL.C	0.176
3	LDL.D	-0.172
4	M.HDL.C	-0.137
5	XXL.VLDL.TG	0.117
6	XS.VLDL.TG	-0.102
7	S.VLDL.TG	-0.090
8	Est.C	0.065
9	ApoA1	-0.052
10	Serum.C	-0.010
	ApoB	0
	HDL.C	0
	HDL.D	0
	IDL.C	0
	IDL.TG	0
	L.VLDL.C	0
	L.VLDL.TG	0
	LDL.C	0
	M.VLDL.C	0
	M.VLDL.TG	0
	S.HDL.TG	0
	S.LDL.C	0
	S.VLDL.C	0
	Serum.TG	0
	SM	0
	Tot.FA	0
	TotPG	0
	VLDL.D	0
	XL.HDL.TG	0
	XL.VLDL.TG	0

Supplementary Table 15: Ranking of risk factors for age-related macular degeneration using Elastic Net regression (L1+L2 penalty) according to their regularised causal effect estimate after excluding genetic variants in the *LIPC, FUT2* and *APOE* region $n = 145$. Abbreviations: beta L1+L2=L1 and L2 regularised causal effect.

3 Supplementary Methods

Derivation of Bayes factors for a set of risk factors

In this note, we derive a closed form expression for the Bayes factor that quantifies the evidence for a particular model (one risk factor or set ofmultiple risk factors) to have a causal effect on the outcome compared to the Null model, which includes no risk factor and no intercept.

Building on the 2-sample MR approach [1] our work is based on summarised data, where genetic variants are used as instrumental variables. In univariable MR, we observe for each genetic variant $i = 1, ..., n$ the association of variant i with the risk factor $\mathbf{X}$ measured by the beta-coefficient β_{X_i} from a univariable regression where the genetic variant i is regressed on the risk factor $\mathbf{X}$, and the association of variant i with the outcome $\mathbf{Y}$ measured by the beta-coefficient β_{Y_i} where the genetic variant i is regressed on the outcome $\mathbf{Y}$, respectively. The causel effect θ of risk factor $\mathbf{X}$ on $\mathbf{Y}$ can be estimated using the IVW estimate or equivalently the following weighted regression without an intercept

$$\beta_{Y_i} = \theta\beta_{X_i} + \epsilon_i, \quad \epsilon_i \sim \mathcal{N}(0, \text{se}(\beta_{Y_i})^2). \tag{1}$$

The same causal effect θ can be derived using a 2-stage least squares approach [2]. In fact, the beta-coefficients are estimates of the genetic association, but we omit the "hat" notation and treat the beta-coefficient as observations. A further assumption for this approach is that the genetic variants are independent (or uncorrelated) which can be controlled in the selection process of the genetic variants. Extension for correlated variants are for example described in [2].

In order to consider multiple risk factors jointly in one model multivariable MR was introduced in [3]. In the following, we consider $j = 1, ..., d$ risk factors. Assume $\boldsymbol{\beta}_{\mathbf{X}} = \{\boldsymbol{\beta}_{X_1}, ..., \boldsymbol{\beta}_{X_d}\}$ to be a matrix of dimension $n \times d$, where d is the number of risk factors and n is the number of genetic variants. Again each individual element $\beta_{X_{i,j}}$ of the predictor matrix is derived from a univariable regression where the genetic variant i is regressed on the risk factor X_j. Multivariable MR can be cast as a weighted linear multivariable regression model

$$\beta_{Y_i} = \theta_1\beta_{X_{i1}} + \theta_2\beta_{X_{i2}} + \ldots + \theta_d\beta_{X_{id}} + \epsilon_i, \quad \epsilon_i \sim \mathcal{N}(0, \text{se}(\beta_{Y_i})^2), \tag{2}$$

where the dependent variable is the association with the outcome β_Y measured on $i = 1, ..., n$ instrumental variables and the predictors are the $j = 1, ..., d$ genetic associations with the d risk factors. In matrix notation this can be written as

$$\boldsymbol{\beta}_{\mathbf{Y}} = \boldsymbol{\beta}_{\mathbf{X}}\boldsymbol{\theta} + \boldsymbol{\epsilon}, \qquad \boldsymbol{\epsilon} \sim \mathcal{N}(\mathbf{0}, \text{se}(\boldsymbol{\beta}_{\mathbf{Y}})^2). \tag{3}$$

In other words, the risk factors represent the variable space and the genetic variants used as instrumental variables are treated as observations. In practise, we standardise each observation of both, β_{Y_i} and β_{X_i} by dividing by $se(\beta_{Y_i})$ before the analysis and we assume in the following derivations that $\boldsymbol{\beta}_{\mathbf{Y}}$ and $\boldsymbol{\beta}_{\mathbf{X}}$ are standardised.

We use Bayes factors [4] in order to quantify the evidence for a particular model. By model we refer to the set of risk factors which have a causal effect on the outcome of interest. In order to formalise which risk factors are part of a specific model $M_{\boldsymbol{\gamma}}$ and consequently have a causal effect on the outcome we introduce a binary indicator $\boldsymbol{\gamma}$ of length d that indicates which risk factors are selected and which ones are not

$$\gamma_j = \begin{cases} 1, & \text{if the } j\text{th risk factor is selected,} \\ 0 & \text{otherwise.} \end{cases} \tag{4}$$

The indicator $\boldsymbol{\gamma}$ encodes a specific regression model $M_{\boldsymbol{\gamma}}$ that includes the risk factors as indicated in $\boldsymbol{\gamma}$. Accordingly, we define $\boldsymbol{\beta}_{\mathbf{X}_{\boldsymbol{\gamma}}}$ as the design matrix of the risk factors included and $\boldsymbol{\theta}_{\boldsymbol{\gamma}}$ as the respective causal effects.

The computation of the Bayes factor for model $M_{\boldsymbol{\gamma}}$ against the Null model M_0, i.e. including no risk factor and no intercept, as presented in the Methods section of the main article requires two ingredients: First the marginal probability of $\boldsymbol{\beta}_{\mathbf{Y}}$ given $\boldsymbol{\beta}_{\mathbf{X}_{\boldsymbol{\gamma}}}$ of model $M_{\boldsymbol{\gamma}}$ and second, the marginal probability of $\boldsymbol{\beta}_{\mathbf{Y}}$ given the Null model M_0, which we derive as follows:

1. $p_{\boldsymbol{\gamma}}(\boldsymbol{\beta}_{\mathbf{Y}} \mid \boldsymbol{\beta}_{\mathbf{X}_{\boldsymbol{\gamma}}})$: the marginal probability of $\boldsymbol{\beta}_{\mathbf{Y}}$ given $\boldsymbol{\beta}_{\mathbf{X}_{\boldsymbol{\gamma}}}$

   In order to model the correlation between risk factors we base our likelihood on a multivariate Gaussian distribution

$$\boldsymbol{\beta}_{\mathbf{Y}} \mid \boldsymbol{\beta}_{\mathbf{X}_{\boldsymbol{\gamma}}}, \boldsymbol{\theta}_{\boldsymbol{\gamma}}, \tau \sim N(\boldsymbol{\beta}_{\mathbf{X}_{\boldsymbol{\gamma}}} \boldsymbol{\theta}_{\boldsymbol{\gamma}}, \frac{1}{\tau}). \tag{5}$$

   Following Servin and Stephens' D_2 prior [5] we use the following conjugate prior assumptions for the causal effects $\boldsymbol{\theta}_{\boldsymbol{\gamma}}$, the residual ϵ and the precision τ

$$\begin{aligned} \boldsymbol{\theta}_{\boldsymbol{\gamma}} \mid \tau &\sim N(0, \boldsymbol{\nu}/\tau), \\ \epsilon &\sim N(0, \frac{1}{\tau}), \\ \tau &\sim \Gamma(\kappa/2, \lambda/2), \end{aligned} \tag{6}$$

   where $A \mid B$ is defined as A conditional on B. Further we can derive analytically the joint posterior distribution for $\boldsymbol{\theta}_{\boldsymbol{\gamma}}$ and τ as

$$\begin{aligned} \tau \mid \boldsymbol{\beta}_{\mathbf{Y}}, \boldsymbol{\beta}_{\mathbf{X}_{\boldsymbol{\gamma}}} &\sim \Gamma((n+\kappa)/2, 1/2(\boldsymbol{\beta}_Y^t \boldsymbol{\beta}_Y - \boldsymbol{\Theta}^{\mathbf{t}} \boldsymbol{\Omega}^{\mathbf{-1}} \boldsymbol{\Theta} + \lambda)), \\ \boldsymbol{\theta}_{\boldsymbol{\gamma}} \mid \boldsymbol{\beta}_{\mathbf{Y}}, \boldsymbol{\beta}_{\mathbf{X}_{\boldsymbol{\gamma}}}, \tau &\sim N(\boldsymbol{\Theta}, \frac{1}{\tau} \boldsymbol{\Omega}), \end{aligned}$$

   where

$$\underbrace{\boldsymbol{\Theta}}_{d\times 1} = \underbrace{\boldsymbol{\Omega}}_{d\times d} \underbrace{\boldsymbol{\beta}_{\mathbf{X}_{\boldsymbol{\gamma}}}{}^t}_{d\times n} \underbrace{\boldsymbol{\beta}_{\mathbf{Y}}}_{n\times 1}, \tag{7}$$

$$\boldsymbol{\Omega} = \underbrace{(\boldsymbol{\nu}^{-1} + \boldsymbol{\beta}_{\mathbf{X}_{\boldsymbol{\gamma}}}^{\mathbf{t}} \boldsymbol{\beta}_{\mathbf{X}_{\boldsymbol{\gamma}}})^{-1}}_{d\times d}. \tag{8}$$

Next we integrate out the causal effects $\boldsymbol{\theta_\gamma}$. To begin with, we sort the equation so that the integral contains only terms dependent on $\boldsymbol{\theta_\gamma}$

$$
\begin{aligned}
p_{\boldsymbol{\gamma}}(\boldsymbol{\beta}_{\mathbf{Y}} \mid \boldsymbol{\beta}_{\mathbf{X}_{\boldsymbol{\gamma}}}, \tau) &= \int_{-\infty}^{\infty} \frac{p_{\boldsymbol{\gamma}}(\boldsymbol{\beta}_{\mathbf{Y}} \mid \boldsymbol{\beta}_{\mathbf{X}_{\boldsymbol{\gamma}}}, \boldsymbol{\theta}_{\boldsymbol{\gamma}}, \tau) p_{\boldsymbol{\gamma}}(\boldsymbol{\theta}_{\boldsymbol{\gamma}} \mid \tau)}{p_{\boldsymbol{\gamma}}(\boldsymbol{\theta}_{\boldsymbol{\gamma}} \mid \boldsymbol{\beta}_{\mathbf{Y}}, \boldsymbol{\beta}_{\mathbf{X}_{\boldsymbol{\gamma}}}, \tau)} \delta\boldsymbol{\theta}_{\boldsymbol{\gamma}} \\
&= \int_{-\infty}^{\infty} \frac{(2\pi)^{-\frac{n}{2}} \tau^{\frac{n}{2}} \exp\left\{-\frac{\tau}{2}(\boldsymbol{\beta}_{\mathbf{Y}} - \boldsymbol{\beta}_{\mathbf{X}_{\boldsymbol{\gamma}}}\boldsymbol{\theta}_{\boldsymbol{\gamma}})^t(\boldsymbol{\beta}_{\mathbf{Y}} - \boldsymbol{\beta}_{\mathbf{X}_{\boldsymbol{\gamma}}}\boldsymbol{\theta}_{\boldsymbol{\gamma}})\right\}}{(2\pi)^{-\frac{1}{2}} \frac{\|\boldsymbol{\Omega}\|^{-1/2}}{\|\tau\|^{-1/2}} \exp\left\{-\frac{\tau}{2}(\boldsymbol{\theta}_{\boldsymbol{\gamma}} - \boldsymbol{\Theta})^t\boldsymbol{\Omega}^{-1}(\boldsymbol{\theta}_{\boldsymbol{\gamma}} - \boldsymbol{\Theta})\right\}} \\
&\quad \times (2\pi)^{-\frac{1}{2}} \frac{\|\boldsymbol{\nu}\|^{-1/2}}{\|\tau\|^{-1/2}} \exp\left\{-\frac{\tau}{2\boldsymbol{\nu}} \boldsymbol{\theta}_{\boldsymbol{\gamma}}^{\mathbf{t}} \boldsymbol{\theta}_{\boldsymbol{\gamma}}\right\} \delta\boldsymbol{\theta}_{\boldsymbol{\gamma}} \\
&= (2\pi)^{-\frac{n}{2}} \tau^{\frac{n}{2}} \frac{\|\boldsymbol{\Omega}\|^{1/2}}{\|\nu\|^{1/2}} \exp\left\{-\frac{\tau}{2}(\boldsymbol{\beta}_{\mathbf{Y}}{}^t\boldsymbol{\beta}_{\mathbf{Y}} - \boldsymbol{\Theta}^{\mathbf{t}}\boldsymbol{\Omega}^{-\mathbf{1}}\boldsymbol{\Theta})\right\} \\
&\quad \times \int_{-\infty}^{\infty} \exp\left\{-\frac{\tau}{2}\left(-2\boldsymbol{\theta}_{\boldsymbol{\gamma}}^{\mathbf{t}}\boldsymbol{\beta}_{\mathbf{X}_{\boldsymbol{\gamma}}}^{\mathbf{t}}\boldsymbol{\beta}_{\mathbf{Y}} + \boldsymbol{\theta}_{\boldsymbol{\gamma}}^{\mathbf{t}}(\boldsymbol{\beta}_{\mathbf{X}_{\boldsymbol{\gamma}}}^{\mathbf{t}}\boldsymbol{\beta}_{\mathbf{X}_{\boldsymbol{\gamma}}} - \boldsymbol{\nu}^{-1})\boldsymbol{\theta}_{\boldsymbol{\gamma}} - \right.\right. \\
&\qquad\qquad \left.\left. \boldsymbol{\theta}_{\boldsymbol{\gamma}}^{\mathbf{t}}\boldsymbol{\Omega}^{-\mathbf{1}}\boldsymbol{\theta}_{\boldsymbol{\gamma}} + 2\boldsymbol{\theta}_{\boldsymbol{\gamma}}^{\mathbf{t}}\boldsymbol{\Omega}^{-\mathbf{1}}\boldsymbol{\Theta}\right)\right\} \delta\boldsymbol{\theta}_{\boldsymbol{\gamma}},
\end{aligned}
$$

where $\|\mathbf{X}\|$ denotes the determinant of a matrix $\mathbf{X}$ and ∞ infinity. Note that the final line in the integral can be simplified to

$$
-2\boldsymbol{\theta}_{\boldsymbol{\gamma}}^{\mathbf{t}}(\mathbf{A} - \mathbf{D}) + \boldsymbol{\theta}_{\boldsymbol{\gamma}}^{\mathbf{t}}(\mathbf{B} - \mathbf{C})\boldsymbol{\theta}_{\boldsymbol{\gamma}}, \tag{9}
$$

where

$$
\begin{aligned}
\mathbf{A} &= \boldsymbol{\beta}_{\mathbf{X}_{\boldsymbol{\gamma}}}^{\mathbf{t}}\boldsymbol{\beta}_{\mathbf{Y}} \\
\mathbf{B} &= (\boldsymbol{\beta}_{\mathbf{X}_{\boldsymbol{\gamma}}}^{\mathbf{t}}\boldsymbol{\beta}_{\mathbf{X}_{\boldsymbol{\gamma}}} - \boldsymbol{\nu}^{-1}) \\
\mathbf{C} &= \boldsymbol{\Omega}^{-\mathbf{1}} \\
\mathbf{D} &= \boldsymbol{\Omega}^{-\mathbf{1}}\boldsymbol{\Theta}
\end{aligned} \tag{10}
$$

By completing the square in $\boldsymbol{\theta_\gamma}$ and integrating out $\boldsymbol{\theta_\gamma}$ the final integral equals 1.

Overall, this simplifies to

$$
p_{\boldsymbol{\gamma}}(\beta_Y \mid \boldsymbol{\beta}_{\mathbf{X}_{\boldsymbol{\gamma}}}, \tau) = (2\pi)^{-\frac{n}{2}} \tau^{\frac{n}{2}} \frac{\|\boldsymbol{\Omega}\|^{1/2}}{\|\boldsymbol{\nu}\|^{1/2}} \exp\left\{-\frac{\tau}{2}(\boldsymbol{\beta}_{\mathbf{Y}}{}^t\boldsymbol{\beta}_{\mathbf{Y}} - \boldsymbol{\Theta}^{\mathbf{t}}\boldsymbol{\Omega}^{-\mathbf{1}}\boldsymbol{\Theta})\right\} \tag{11}
$$

Next we integrate out the precision τ

$$
\begin{aligned}
p_{\boldsymbol{\gamma}}(\boldsymbol{\beta}_Y \mid \boldsymbol{\beta}_{\mathbf{X}_{\boldsymbol{\gamma}}}) &= \int_0^{\infty} p_{\boldsymbol{\gamma}}(\boldsymbol{\beta}_{\mathbf{Y}} \mid \boldsymbol{\beta}_{\mathbf{X}_{\boldsymbol{\gamma}}}, \tau) p(\tau) \delta\tau \qquad (12) \\
&= (2\pi)^{-\frac{n}{2}} \frac{\|\boldsymbol{\Omega}\|^{1/2}}{\|\boldsymbol{\nu}\|^{1/2}} \\
&\quad \times \int_0^{\infty} \tau^{\frac{(n+\kappa)}{2}-1} \exp\left\{-\frac{1}{2}(\boldsymbol{\beta}_{\mathbf{Y}}{}^t\boldsymbol{\beta}_{\mathbf{Y}} - \boldsymbol{\Theta}^{\mathbf{t}}\boldsymbol{\Omega}^{-\mathbf{1}}\boldsymbol{\Theta} + \lambda)\tau\right\} \delta\tau.
\end{aligned}
$$

The above integral is the normalisation constant of a Gamma distribution with shape $\alpha = \frac{(n+\kappa)}{2}$ and rate $\beta = \frac{1}{2}(\boldsymbol{\beta_Y}^t\boldsymbol{\beta_Y} - \mathbf{\Theta^t\Omega^{-1}\Theta} + \lambda)$. Thus the above simplifies exactly to

$$p_{\boldsymbol{\gamma}}(\boldsymbol{\beta_Y} \mid \boldsymbol{\beta_{X_\gamma}}) = (2\pi)^{-\frac{n}{2}} \frac{\|\mathbf{\Omega}\|^{1/2}}{\|\boldsymbol{\nu}\|^{1/2}} (\tfrac{\lambda}{2})^{\frac{\kappa}{2}} \frac{\Gamma(\frac{n+\kappa}{2})}{\Gamma(\frac{\kappa}{2})} \tag{13}$$

$$\times \left\{\tfrac{1}{2}(\boldsymbol{\beta_Y}^t\boldsymbol{\beta_Y} - \mathbf{\Theta^t\Omega^{-1}\Theta} + \lambda)\right\}^{\frac{-(n+\kappa)}{2}}. \tag{14}$$

2. $p_0(\boldsymbol{\beta_Y})$: the marginal probability of $\boldsymbol{\beta_Y}$ given the Null model M_0

   Next, we derive the marginal probability of the Null model, i.e. where no risk factor and no intercept is included. Under the Null we assume

$$\beta_Y \mid \frac{1}{\tau} \sim N(0, \frac{1}{\tau}) \tag{15}$$

   with an expectation fixed at zero, which is a consequence of the missing intercept.

   First, we integrate out the precision τ

$$\begin{aligned} p_0(\boldsymbol{\beta_Y}) &= \int_0^\infty p_0(\boldsymbol{\beta_Y} \mid \tau)p(\tau)\delta\tau \\ &= (2\pi)^{-\frac{n}{2}} \int_0^\infty \tau^{\frac{(n+\kappa)}{2}-1} \exp\left\{-\frac{1}{2}(\boldsymbol{\beta_Y}^t\boldsymbol{\beta_Y} + \lambda)\tau\right\} \delta\tau. \end{aligned} \tag{16}$$

   Again the above integral is the normalisation constant of a Gamma distribution with shape $\alpha = \frac{(n+\kappa)}{2}$ and rate $\beta_0 = \frac{1}{2}(\boldsymbol{\beta_Y}^t\boldsymbol{\beta_Y} + \lambda)$. Thus the above simplifies to

$$p_0(\boldsymbol{\beta_Y}) = (2\pi)^{-\frac{n}{2}} (\frac{\lambda}{2})^{\frac{\kappa}{2}} \frac{\Gamma(\frac{n+\kappa}{2})}{\Gamma(\frac{\kappa}{2})} \left(\frac{1}{2}(\boldsymbol{\beta_Y}^t\boldsymbol{\beta_Y} + \lambda)\right)^{-\frac{(n+\kappa)}{2}}. \tag{17}$$

The Bayes factor for model $M_{\boldsymbol{\gamma}}$ against M_0 is defined as the ratio of the marginal probability of $\boldsymbol{\beta_Y}$ given model $M_{\boldsymbol{\gamma}}$ (13) over the marginal probability of $\boldsymbol{\beta_Y}$ given the Null model (17)

$$\begin{aligned} BF(M_{\boldsymbol{\gamma}}) &= \frac{p_{\boldsymbol{\gamma}}(\boldsymbol{\beta_Y} \mid \boldsymbol{\beta_{X_\gamma}})}{p_0(\boldsymbol{\beta_Y})} \\ &= \frac{\frac{\|\mathbf{\Omega}\|^{1/2}}{\|\boldsymbol{\nu}\|^{1/2}} \left(\frac{1}{2}(\boldsymbol{\beta_Y}^t\boldsymbol{\beta_Y} - \mathbf{\Theta^t\Omega^{-1}\Theta} + \lambda)\right)^{-(n+\kappa)/2}}{\left(\frac{1}{2}(\boldsymbol{\beta_Y}^t\boldsymbol{\beta_Y} + \lambda)\right)^{-(n+\kappa)/2}} \\ &= \frac{\|\mathbf{\Omega}\|^{1/2}}{\|\boldsymbol{\nu}\|^{1/2}} \left(\frac{\boldsymbol{\beta_Y}^t\boldsymbol{\beta_Y} - \mathbf{\Theta^t\Omega^{-1}\Theta} + \lambda}{\boldsymbol{\beta_Y}^t\boldsymbol{\beta_Y} + \lambda}\right)^{-(n+\kappa)/2}. \end{aligned} \tag{18}$$

Next we consider the limit as κ, $\lambda \to 0$. κ and λ are the shape and scale parameter of the Gamma Distribution, which is the conjugate distribution for

precision. In the limiting case the expectation of the error precision would converge towards a point mass at zero. A precision that converges to zero translates into an error variance that converges to infinity. Thus the limiting case represents a dominant error noise and no variance explained by the model, which is a conservative prior assumption. Moreover, the limit $\lambda \to 0$ leads to the invariance property of the posterior for $\boldsymbol{\theta}$, ie the posterior of $\boldsymbol{\theta}$ changes appropriately with shifts and scaling (for example inverse-variance weighting) operations on $\boldsymbol{\beta}_{\mathbf{Y}}$.

In limit, the Bayes Factor simplifies to the following closed form expression

$$BF(M_{\boldsymbol{\gamma}}) = \frac{\|\boldsymbol{\Omega}\|^{1/2}}{\|\boldsymbol{\nu}\|^{1/2}} \left(\frac{\boldsymbol{\beta}_{\mathbf{Y}}{}^t \boldsymbol{\beta}_{\mathbf{Y}} - \boldsymbol{\Theta}^{\mathbf{t}} \boldsymbol{\Omega}^{\mathbf{-1}} \boldsymbol{\Theta}}{\boldsymbol{\beta}_{\mathbf{Y}}{}^t \boldsymbol{\beta}_{\mathbf{Y}}} \right)^{-n/2} . \tag{19}$$

These Bayes factors are then used in the model averaging to quantify the evidence for a model and together with the prior are used to evaluate which model or set of risk factors has the largest support to have a causal effect on the outcome.

References

[1] Pierce, B. L. & Burgess, S. Efficient Design for Mendelian Randomization Studies: Subsample and 2-Sample Instrumental Variable Estimators. *American Journal of Epidemiology* **178**, 1177–1184 (2013). URL http://dx.doi.org/10.1093/aje/kwt084.

[2] Burgess, S., Dudbridge, F. & Thompson, S. G. Combining information on multiple instrumental variables in Mendelian randomization: comparison of allele score and summarized data methods. *Statistics in medicine* **35**, 1880–1906 (2016). URL https://www.ncbi.nlm.nih.gov/pubmed/26661904.

[3] Burgess, S. & Thompson, S. G. Multivariable Mendelian randomization: the use of pleiotropic genetic variants to estimate causal effects. *American journal of epidemiology* **181**, 251–260 (2015). URL https://www.ncbi.nlm.nih.gov/pubmed/25632051.

[4] Kass, R. E. & Raftery, A. E. Bayes factors. *Journal of the American Statistical Association* **90**, 773–795 (1995). URL https://amstat.tandfonline.com/doi/abs/10.1080/01621459.1995.10476572.

[5] Servin, B. & Stephens, M. Imputation-based analysis of association studies: candidate regions and quantitative traits. *PLoS Genet* **3**, e114 (2007). URL https://www.ncbi.nlm.nih.gov/pubmed/17676998.
