# Supplementary material for: Selecting likely causal risk factors from high-throughput experiments using multivariable Mendelian randomization
Source: Nat Commun. 2020 Jan 7;11:29. doi: 10.1038/s41467-019-13870-3 (PMC6946691; doi:10.1038/s41467-019-13870-3)
Supplement: Supplementary file 3 — Reporting Summary [file 41467_2019_13870_MOESM3_ESM.pdf]

nature research

Corresponding author(s): Stephen Burgess

Reporting Summary

Nature Research wishes to improve the reproducibility of the work that we publish. This form provides structure for consistency and transparency in reporting. For further information on Nature Research policies, see Authors & Referees and the Editorial Policy Checklist.

Statistical parameters

When statistical analyses are reported, confirm that the following items are present in the relevant location (e.g. figure legend, table legend, main text, or Methods section).

n/a	Confirmed	
☐	☒	The exact sample size (*n*) for each experimental group/condition, given as a discrete number and unit of measurement
☐	☒	An indication of whether measurements were taken from distinct samples or whether the same sample was measured repeatedly
☒	☐	The statistical test(s) used AND whether they are one- or two-sided<br>*Only common tests should be described solely by name; describe more complex techniques in the Methods section.*
☒	☐	A description of all covariates tested
☐	☒	A description of any assumptions or corrections, such as tests of normality and adjustment for multiple comparisons
☐	☒	A full description of the statistics including central tendency (e.g. means) or other basic estimates (e.g. regression coefficient) AND variation (e.g. standard deviation) or associated estimates of uncertainty (e.g. confidence intervals)
☒	☐	For null hypothesis testing, the test statistic (e.g. F, t, r) with confidence intervals, effect sizes, degrees of freedom and P value noted<br>*Give P values as exact values whenever suitable.*
☐	☒	For Bayesian analysis, information on the choice of priors and Markov chain Monte Carlo settings
☒	☐	For hierarchical and complex designs, identification of the appropriate level for tests and full reporting of outcomes
☒	☐	Estimates of effect sizes (e.g. Cohen's d, Pearson's r), indicating how they were calculated
☐	☒	Clearly defined error bars<br>*State explicitly what error bars represent (e.g. SD, SE, CI)*

Our web collection on statistics for biologists may be useful.

Software and code

Policy information about availability of computer code

Data collection	R version 3.4.2 (2017-09-28) -- "Short Summer"<br>Copyright (C) 2017 The R Foundation for Statistical Computing<br>Platform: x86_64-apple-darwin15.6.0 (64-bit)<br><br>Python 2.7.10
Data analysis	R version 3.4.2 (2017-09-28) -- "Short Summer"<br>Copyright (C) 2017 The R Foundation for Statistical Computing<br>Platform: x86_64-apple-darwin15.6.0 (64-bit)<br><br>MR-BMA and publicly available summary data on AMD and NMR metabolites as presented in the applied example is public on https://github.com/verena-zuber/demo_AMD

For manuscripts utilizing custom algorithms or software that are central to the research but not yet described in published literature, software must be made available to editors/reviewers upon request. We strongly encourage code deposition in a community repository (e.g. GitHub). See the Nature Research guidelines for submitting code & software for further information.

Data

Policy information about availability of data

All manuscripts must include a data availability statement. This statement should provide the following information, where applicable:

- Accession codes, unique identifiers, or web links for publicly available datasets
- A list of figures that have associated raw data
- A description of any restrictions on data availability

Our study is based on publicly available summarized data. We reference first the publications that describe in full detail the data generation and second the web resources we used to obtain the data. In particular, this is:

1. Summarized data on genetic associations with NMR metabolites
http://computationalmedicine.fi/data#NMR_GWAS
2. Summarized data on genetic associations with age-related macular degeneration:
http://csg.sph.umich.edu/abecasis/public/amd2015/
3. Summarized data on genetic associations with blood traits
https://www.ebi.ac.uk/gwas/

All data used in our study is in the public domain.

Field-specific reporting

Please select the best fit for your research. If you are not sure, read the appropriate sections before making your selection.

☒ Life sciences ☐ Behavioural & social sciences ☐ Ecological, evolutionary & environmental sciences

For a reference copy of the document with all sections, see nature.com/authors/policies/ReportingSummary-flat.pdf

Life sciences study design

All studies must disclose on these points even when the disclosure is negative.

Sample size	Sample size was obtained from the original studies (Kettunen, J. et al. Nature Communications 7 (2016) and Fritsche, L. G. et al. Nature Genetics 48, 134 EP – (2015)) that produced the data.
Data exclusions	A detailed description of which metabolites are included into the study is given in the manuscript, section: Data pre-processing and analysis for applied example of age-related macular degeneration. The selection of genetic instruments is detailed in the manuscript section: Simulation results on NMR metabolite data. We used genetic variants associated with blood lipids, in particular triglycerides, LDL and HDL cholesterol, as reported by the Global Lipid Genetics Consortium Nature Genetics 45, 1274 EP – (2013). Exclusion of genetic variants is detailed in the manuscript, section Metabolites as risk factors for age-related macular degeneration.
Replication	N/A
Randomization	N/A
Blinding	N/A

Reporting for specific materials, systems and methods

Materials & experimental systems

n/a	Involved in the study	
☒	☐	Unique biological materials
☒	☐	Antibodies
☒	☐	Eukaryotic cell lines
☒	☐	Palaeontology
☒	☐	Animals and other organisms
☒	☐	Human research participants

Methods

n/a	Involved in the study	
☒	☐	ChIP-seq
☒	☐	Flow cytometry
☒	☐	MRI-based neuroimaging
